# TALENs-mediated gene disruption of FLT3 in leukemia cells: Using genome-editing approach for exploring the molecular basis of gene abnormality

**DOI:** 10.1038/srep18454

**Published:** 2015-12-16

**Authors:** Jue Wang, Tongjuan Li, Mi Zhou, Zheng Hu, Xiaoxi Zhou, Shiqiu Zhou, Na Wang, Liang Huang, Lei Zhao, Yang Cao, Min Xiao, Ding Ma, Pengfei Zhou, Zhen Shang, Jianfeng Zhou

**Affiliations:** 1 Department of hematology, Tongji Hospital, Tongji Medical College, Huazhong University of Science and Technology, Wuhan, Hubei, China; 2 Cancer Biology Research Center, Tongji Hospital, Tongji Medical College, Huazhong University of Science and Technology, Wuhan, Hubei, China; 3 Wuhan YZY Bio-Pharma Co., Ltd., Wuhan, Hubei, China

## Abstract

Novel analytic tools are needed to elucidate the molecular basis of leukemia-relevant gene mutations in the post-genome era. We generated isogenic leukemia cell clones in which the FLT3 gene was disrupted in a single allele using TALENs. Isogenic clones with mono-allelic disrupted FLT3 were compared to an isogenic wild-type control clone and parental leukemia cells for transcriptional expression, downstream FLT3 signaling and proliferation capacity. The global gene expression profiles of mutant K562 clones and corresponding wild-type controls were compared using RNA-*seq*. The transcriptional levels and the ligand-dependent autophosphorylation of FLT3 were decreased in the mutant clones. TALENs-mediated FLT3 haplo-insufficiency impaired cell proliferation and colony formation *in vitro*. These inhibitory effects were maintained *in vivo*, improving the survival of NOD/SCID mice transplanted with mutant K562 clones. Cluster analysis revealed that the gene expression pattern of isogenic clones was determined by the FLT3 mutant status rather than the deviation among individual isogenic clones. Differentially expressed genes between the mutant and wild-type clones revealed an activation of nonsense-mediated decay pathway in mutant K562 clones as well as an inhibited FLT3 signaling. Our data support that this genome-editing approach is a robust and generally applicable platform to explore the molecular bases of gene mutations.

Acute leukemia (AL) is among the most common forms of hematological malignancies and exhibits a striking heterogeneity in genetic makeup and clinical outcome. Despite striking success in treating some subtypes of AL due to progress in the understanding of cytogenetic/gene mutations and the introduction of corresponding targeted therapies, many genetically high-risk AL subtypes remain refractory to current standard regimens and display poor prognosis, as the current therapeutic strategies are insufficient to cure genetically high-risk AL^1^,^2^. The increasing classification/stratification of AL by the World Health Organization (WHO) and National Comprehensive Cancer Network (NCCN) based on genetic orientation highlights the profound impact of cytogenetic/gene mutations on decision-making regarding clinical management. Therefore, there is an urgent need to dissect the molecular basis of AL in these patients and to develop novel treatment strategies.

The advances in high-throughput sequencing technologies enabled the characterization of AL from a global genomic perspective in an unbiased and comprehensive manner rather than a candidate gene approach. The identification of highly recurrent gene mutations in AL has provided invaluable preclinical rationales for improved diagnosis, patient stratification and targeted therapy^3^,^4^. Nevertheless, although exhaustive landscapes of AL genomes are emerging, elucidating how these recurrent gene mutations are associated with clinical phenotypes and treatment outcomes has proven difficult. The ability to determine the clinical relevance of these identified individual genetic abnormalities is greatly needed. More importantly, uncovering the molecular events that are central to leukemia biology is a major challenge in the human genome era and is fundamentally important for the future of genetic medicine^5^.

Several approaches have been widely used to explore the molecular mechanism underlying a given gene mutation. Many elegant leukemia animal models have been established using genetically engineered strategies and have provided persuasive *in vivo* insight into the driving factors of leukemogenesis. In the coming years, genetically engineered animal models will remain among the most reliable tools to study the association between genetic defects and clinical phenotypes, and contribution to the design and development of novel molecular-targeted strategies. Nevertheless, the animal model approach has some intrinsic shortcomings. For example, animal models may not always accurately mimic the leukemia phenotype relevant to the clinical setting^6^, aside from the time-consuming and expensive process of generating and maintaining a transgenic model. Alternatively, cell lines and primary sample-based models have also been widely used to explore the critical molecular events that lead to tumor cell phenotypes. Several factors have limited the identification of actual molecular mechanisms due to the huge discrepancy in genetic backgrounds among primary samples, the non-physiological levels of transgene overexpression/knockdown and the interference introduced by randomly integrated viral vectors. Therefore, novel analytic tools are urgently needed to address the molecular mechanisms underlying leukemia-relevant gene function in the post-genome era.

Transcription activator-like effector (TALE) nucleases (TALENs), an efficient genome editing tool, are artificial fusion proteins containing the catalytic domain of the endonuclease FokI and a designed TALE DNA-binding domain that recognizes a specific DNA sequence^7^,^8^. The binding of two separate TALENs to adjacent DNA sequences enables dimerization of FokI and cleavage of the target DNA, introducing site-specific double-strand breaks (DSBs). Subsequently, cellular DNA repair *via* either homology-directed repair or the non-homologous end joining (NHEJ) pathway is activated^9^. Thus far, genome-editing technology has been successfully applied to induce myeloid malignancy in normal hematopoietic stem cells in mice^10^. However, few studies have performed this technique to investigate the molecular events caused by individual gene abnormality in leukemia.

In this study, we generated isogenic clones, in two individual leukemia cell lines, by disrupting FMS-like tyrosine kinase 3 (FLT3) gene in a single allele using designed TALENs. The resulting isogenic clones which were only different in the FLT3 mutant status were compared for FLT3 downstream signaling, proliferation capacity and transcriptional expression. Our data strongly support that this genome-editing approach can serve as a robust and generally applicable platform for exploring the molecular basis of a given gene abnormality.

## Results

### Generation of isogenic leukemia clones carrying disrupted FLT3 in a single allele using designed TALENs

**Figure 1:**
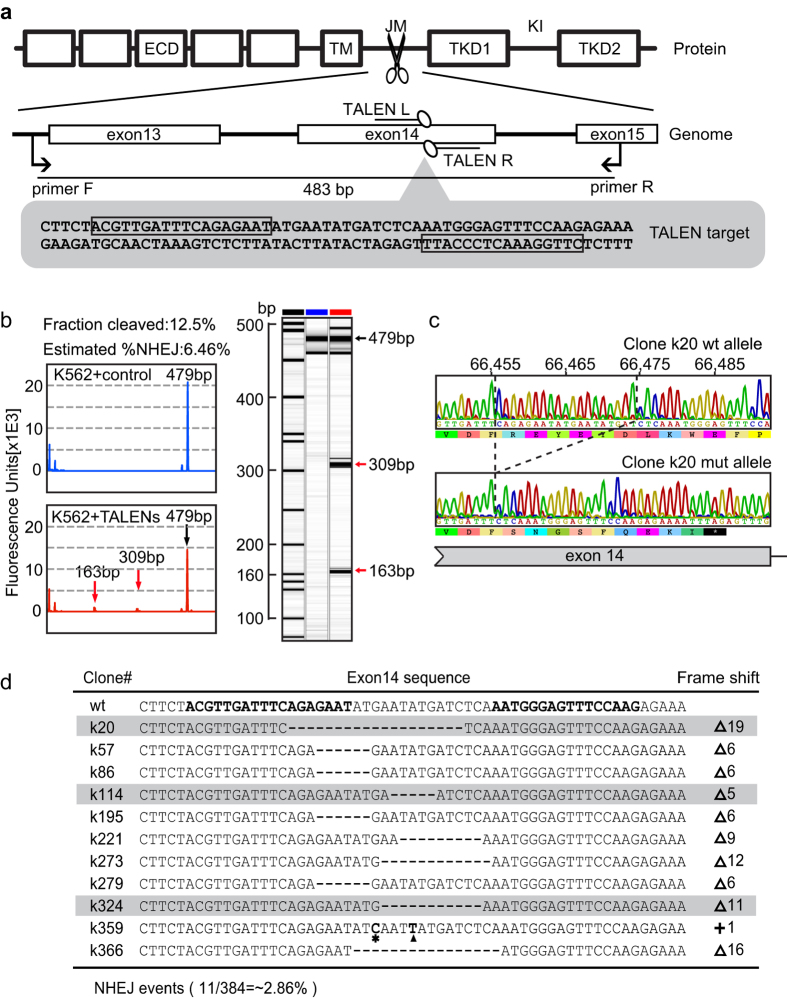
TALEN-mediated gene disruption of FLT3 in the JM domain in leukemia cells. (**a**) Schematic overview of the strategy to disrupt the JM domain of FLT3. The three exons that encode the JM domain, the TALEN pair used (TALEN L and TALEN R) and the primer pair (Primer F and Primer R) for PCR and DNA sequencing to screen the mutant clones were shown. The boxes indicate the TALEN binding sequences in the lower diagram in grey. (**b**) A T7 Endonuclease I assay presented as an electrophoretic trace (the left upper and lower panels) and a pseudo-gel image (the right panel). The blue-colored trace and the lane labeled with a blue bar at the top correspond to K562 cells transfected with the empty TALEN expression vector as a negative control. The red-colored trace and the lane labeled with a red bar at the top correspond to K562 cells transfected with the TALEN pair. The red arrows denote T7EI cleavage products of the appropriate size, and the single black arrow denotes the original uncleaved PCR product in both the electrophoretic trace and the pseudo-gel image. The internal lane standards were labeled with a black bar. (**c**) Representative sequencing results of targeted K562 clones displaying either the wild-type or targeted mutant allele. The original base numbers in the genomic region of the wild-type FLT3 gene were shown. The dotted lines denote the 19bp deletion in the mutant allele. The colored bars below each chromatogram indicate the predicted proteins. The black bar with an asterisk represents the premature termination codon. (**d**) Summary of Sanger sequencing results of positive isogenic mutant clones derived from the K562 cell line transfected with TALENs targeting FLT3 exon14. The TALEN binding sequences are shown in bold letters in the wild-type clone. The deletions are indicated by dashes, and insertion or base substitution is denoted by a black triangle or an asterisk, respectively. ECD, extracellular domain; TM, transmembrane domain; JM, juxtamembrane domain; TKD, tyrosine kinase domain; KI, kinase insert; TALEN, transcription activator-like effector nuclease; WT, wild-type; Mut, mutant; Bp, base pair; NHEJ, non-homologous end joining.

To generate isogenic leukemia clones carrying a disrupted FLT3 juxtamembrane (JM) domain in a single allele, a pair of TALENs targeting exon14 of FLT3 was designed. The TALEN target site was selected within the sequence of exon14, which encodes the JM domain and is located upstream of the tyrosine kinase domains (TKDs) and the kinase insert (KI) domain, such that insertions or deletions caused by NHEJ could result in disruption of the reading frame or the formation of a premature stop codon. We constructed TALENs composed of 17.5 and 15.5 repeats to cleave a site using a 15 bp spacer according to five computationally derived design guidelines as described previously (Fig. 1a)^11^.

Since the transfection efficiency in K562 cells approached over 90% (Supplementary Fig. S1) under the experimental conditions described in METHODS, the T7E1 mismatch sensitive assay was applied to assess the nuclease activity of the designed TALENs at their intended target in K562 cells. As shown in Fig.1b, T7E1 cleavage products of the expected size were detected on both the electrophoretic trace and the pseudo-gel image, displaying an estimated frequency of NHEJ events of up to 6%. These data demonstrated robust genomic editing capacity of the designed TALENs at the JM domain of endogenous FLT3 in leukemia cells, facilitating subsequent screening of leukemia clones carrying genetically disrupted FLT3.

Before establishing isogenic clonal leukemia models, we assessed the basal expression level of FLT3 in a panel of working cell lines representing AML and CML myeloid blast crisis by Western blot (Supplementary Fig. S2). Consistent with previous studies^12^,^13^, FLT3 protein levels in cell lines harboring FLT3-ITD mutations such as MV4-11 and MOLM-13 were generally higher than those cell lines with wild-type FLT3, whereas FLT3 wild-type cell lines expressed FLT3 protein at different levels. Based on transfection efficiencies and cell viabilities after nucleofection, we chose three individual cell lines, K562 and OCI-AML3 with the lowest and the highest basal wild-type FLT3 expression respectively, and Jurkat cells with undetectable FLT3 expression as negative control^14^, to generate isogenic leukemia clones using our designed TALENs. Immunoprecipitation followed by mass spectrometry (Supplementary methods) was performed to address the specificity of FLT3 protein bands in K562 cells (Supplementary Fig. S3 and Supplementary Fig. S4). PCR and Sanger sequencing were conducted to confirm that there was no FLT3-ITD mutation in the three working parental cell lines.

The TALEN-nucleofected leukemia cells were seeded at low density in a 96-well plate for recovery and homogeneous expansion. The clonal cell populations were isolated and screened *via* PCR amplification of genomic DNA and Sanger sequencing to identify indels characteristic of NHEJ (Fig. 1c). In this experiment, among a total of 384 K562 clones screened, 11 clones (~2.86%) contained heterozygous modifications in exon 14, including 5 clones carrying frameshift mutations and 6 clones carrying in-frame deletions in one allele (Fig. 1d). All 5 isogenic K562 clones carrying a frameshift mutation in FLT3 were predicted to contain a premature termination codon, of which three clones (clones k20, k114 and k324) were selected for the subsequent experiments. Similar results were obtained from OCI-AML3 and Jurkat, and 3 clones from OCI-AML3 (clones o35, o166 and o253) and 2 clones from Jurkat (clones j105 and j212) were confirmed with frame shift mutations (Supplementary Table S1) after screening 480 OCI-AML3 clones and 300 Jurkat clones, respectively. Three wild-type clones derived from the same screening procedure for each cell line were randomly selected as negative controls.

To assess the off-target gene effects of the designed TALENs, Paired Target Finder was employed to search for potential off-target sites. The genomic region containing the 20 most likely off-target candidate sites was PCR-amplified and subjected to sequencing analysis. Mutations within two candidate sites were identified in all isogenic K562 subclones examined; however, the parental K562 cells were confirmed to carry the identical mutations (Table 1). Therefore, no off-target events caused by the designed TALENs were detected.

### Single-allelic disruption of FLT3 induces FLT3 haplo-insufficiency in Leukemia cells

**Figure 2:**
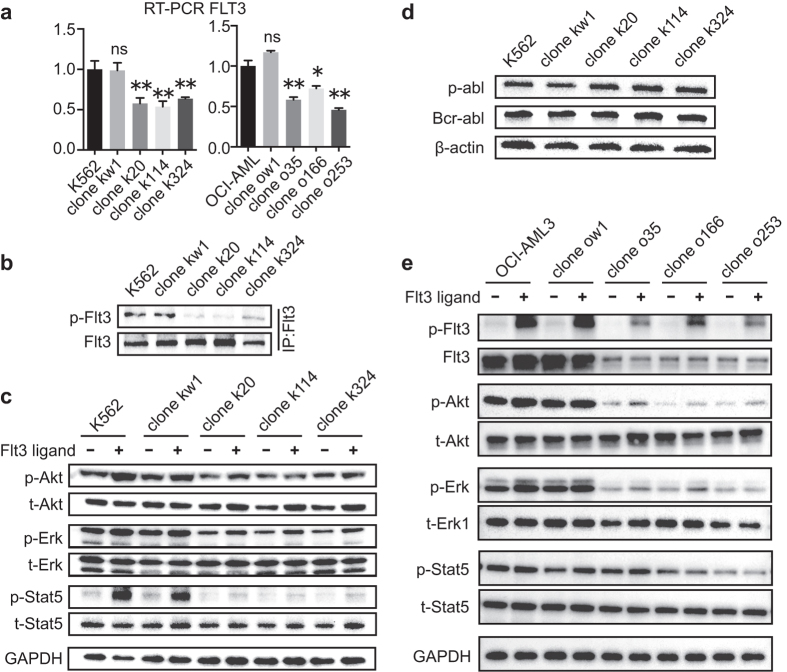
Single-allelic disruption of FLT3 induces FLT3 haplo-insufficiency in leukemia cells. (**a**) Quantitative RT-PCR analysis of the FLT3 transcript levels in K562 and OCI-AML3 clones. The relative expression levels normalized to GAPDH are expressed as the means ± SEM. NS, not significant; *P < 0.05; **P < 0.01 compared to those of the parental cells. (**b**) Representative image of immunoblot analysis of the FLT3 autophosphorylation levels in K562 cells. (**c**) Representative image of immunoblot analysis of the signaling mediators downstream of FLT3 in K562. The cells were pre-starved of growth factors for 12 h and were subsequently exposed to the FLT3 ligand for 10 min before Western blot analysis. (**d**) The activation and expression of bcr-abl in parental K562 cells and the isogenic K562 clones were analyzed by Western blot. (**e**) Representative image of immunoblot analysis of the FLT3 autophosphorylation levels and the signaling mediators downstream of FLT3 in OCI-AML3. IP, immunoprecipitation.

To determine whether the premature termination codon caused by NHEJ mutation in the mutant clones of each cell lines induces FLT3 haplo-insufficiency, we first examined the FLT3 mRNA transcript levels in the parental cell line and its isogenic clones *via* real-time RT-PCR. As expected, significantly lower transcript levels of FLT3 were detected in the mutant clones compared to those of wild-type clones or the parental cell population for both K562 and OCI-AML3 (Fig.2a), implying that premature stop codon-mediated mRNA decay might occur in the mutant FLT3 clones. Next, we sought to determine whether the NHEJ mutation induced a reduced level of FLT3 autophosphorylation upon exposure to treatment of the isogenic mutant clones with FLT3 ligand (FL). Since the K562 cells express the FLT3 protein at a very low level, immunoblotting was performed following immunoprecipitation with FLT3 antibody to examine the potential FL-dependent activity of FLT3 in the K562 clones. As shown in Fig.2b, western blot analyses revealed substantially attenuated FL-dependent autophosphorylation of FLT3 in the mutant clones compared to the wild-type K562 clones, although the reduction of total FLT3 protein level was not significant after immunoprecipitation. The target genes downstream of the FLT3 signaling pathway were further examined *via* Western blot. Although consistent activation of FLT3 target genes was detected in a FL-dependent manner in all K562 clones, the FL-induced phosphorylation level was substantially reduced in the mutant K562 clones compared to their wild-type counterparts (Fig. 2c). Because K562 cells carry the oncogenic bcr/abl fusion gene, which is also a potent activator of the downstream pathways described above^15^, we sought to determine whether the TALENs exerted off-target effects on the bcr/abl protein. As shown in Fig. 2d, neither the basal nor phosphorylated level of bcr/abl was affected by the TALENs procedure, indicating that FLT3 haplo-insufficiency was specifically caused by the introduced mutations. A set of similar immunoblotting assays was performed to validate the TALENs-mediated FLT3 haplo-insufficiency for OCI-AML3 clones (Fig. 2e). As results, a significant down-regulation of FLT3 protein expression and ligand-dependent FLT3 autophosphorylation in the mutant OCI-AML3 clones was easily detected using standard immunoblotting, whereas similar trends of weakened Akt, Erk and Stat5 signaling were observed. However, no such alterations regarding the FLT3 protein expression or downstream signaling could be detected in the negative control Jurkat cells and its isogenic clones (Supplementary Fig. S5). To confirm that the changes in cellular signaling were caused by FLT3 gene disruption, siRNA knockdown was performed in K562, OCI-AML3 and THP-1 (Supplementary methods). As shown in Supplementary Fig. S6, similar alterations in FLT3 signaling occurred 48 hours after siRNA transfection. Collectively, these results demonstrate that TALEN-mediated disruption of exon14 of FLT3 induces FLT3 haplo-insufficiency as indicated by reduced FLT3 autophosphorylation and substantially attenuated FLT3 signaling.

### TALEN-mediated FLT3 haplo-insufficiency impairs cell proliferation and colony forming capacity *in vitro*

**Figure 3:**
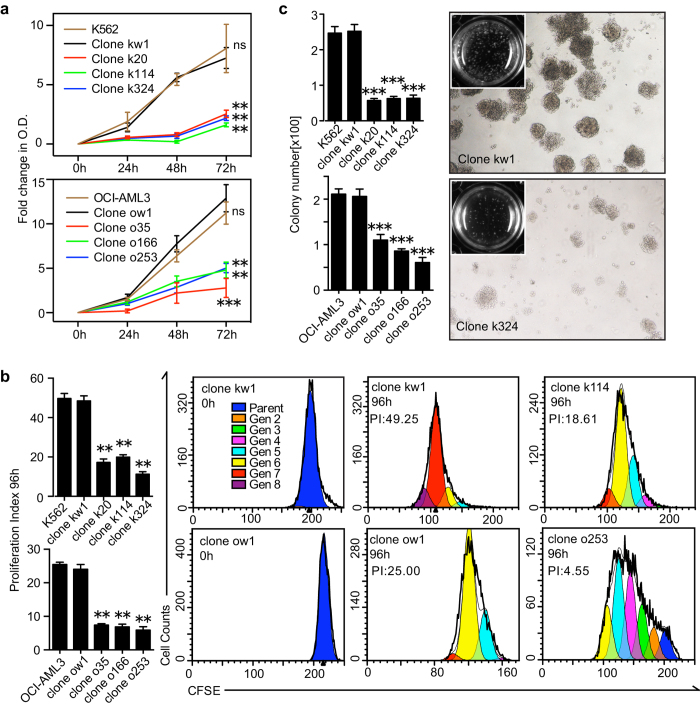
The FLT3 haplo-insufficient clones displayed impaired cell proliferation and colony forming capacity. (**a**) Growth curves of the parental cell lines and its isogenic clones were detected *via* the CCK-8 assay. All OD values were expressed as the fold changes compared to those of the 0 h time point. (**b**) The cells were stained with CFSE and cultured for an additional 4 days. The number of cells in each generation was estimated by deconvolution of the FACS data. Representative modeled generational subsets (colored curves; Gen 2 to 8, generation 2 to 8) are shown, and the proliferation index (PI) was calculated using ModiFit software. Approximately ten thousand cells were analyzed per condition. (**c**) The colony-forming assay in methylcellulose complete medium. Quantitative analyses of the colony numbers are shown in the left panel. Representative results from the wild-type and mutant K562 clones are shown in the right panel. For statistical significance, the values were expressed as the means ± SEM of three independent experiments. *P < 0.05; **P < 0.01; ***P < 0.001.

It is well established that one of the most prominent characteristics of activated FLT3 signaling is the promotion of cell proliferation^16^. Therefore, we sought to investigate the biologic effect of TALEN-mediated FLT3 haplo-insufficiency on leukemia cell proliferation *in vitro*. Based on the CCK8 (Fig. 3a) and CFSE (Fig. 3b) assays in both K562 and OCI-AML3 cells, the proliferation capacity in the mutant FLT3 clones was consistently reduced to nearly 50% or less than those of the corresponding wild-type FLT3 clones, while no inhibition on the proliferation capacity could be observed in the mutant Jurkat clones compared to their wild-type counterparts (Supplementary Fig. S7). In the same experiments, no significant difference in cell proliferation capacity was detected between the isogenic mutant clones (*P* > 0.05). It was also to our surprise that even in K562 cells that express FLT3 at a very low level, the TALEN-mediated FLT3 haplo-insufficiency resulted in significantly impaired proliferation capacity. To rule out the possibility that TALEN-mediated growth inhibition might be an artefact caused by multiple experimental procedures, we generated and sequence-verified an artificial heterozygous FLT3-ITD model (clone KI-H6) in K562 using the same pair of TALENs by homologous directed repair adopting similar protocols as previously reported (Supplementary Figure S8)^17^. By comparing growth capacities of three clones with different FLT3 mutant status (FLT3-wt/wt, wt/-, wt/ITD), we found that the FLT3-ITD knock-in clone (KI-H6) display similar proliferative patterns to that of FLT3 wt/wt clones in the presence of serum (Supplementary Figure S8). The findings support that the growth inhibition on FLT3 wt/- clones was specifically due to FLT3 gene disruption rather than a non-specific artefact caused by the multi-experimental procedures. In the present study, each data point represents the mean of triplicate samples. For each individual assay, the cells were maintained in serial passage culture for at least 2 weeks, indicating a persistent and stable suppressive effect on cell proliferation of heritable FLT3 haplo-insufficiency. In accordance with the results of the CCK8 and CFSE assays, the colony-forming capacity of the isogenic clones was decreased by approximately 75% (p < 0.001), 50% (p < 0.001) and less than 10% (p > 0.05) for K562, OCI-AML3 and Jurkat, respectively, compared to the parental cell line and its wild-type subclones, further confirming that the inhibitory effect of TALENs was FLT3-dependant (Fig. 3c and Supplementary Fig. S7). Next, we used Sorafenib and Quizartinib (AC220) to test whether this growth inhibition could be reproduced by tyrosine kinase inhibitors (TKIs) (Supplementary Fig. S9 and Supplementary methods). As expected, K562 and OCI-AML3 were unresponsive to FLT3 inhibitors within the dosage range reported elsewhere^18^,^19^, and cell toxicity was observed when the TKI concentration was substantially raised. These data validated K562 cells as a robust ‘negative control’ in FLT3 tyrosine kinase inhibitor studies. The profound proliferative inhibition brought out by FLT3-specific TALENs in TKI-resistant cell-lines implied a more potent and persistent interference on FLT3 signaling caused by genome editing approaches.

### TALEN-mediated FLT3 haplo-insufficiency reduces leukemia cell proliferation and improves the survival of NOD/SCID xenografted mice

**Figure 4:**
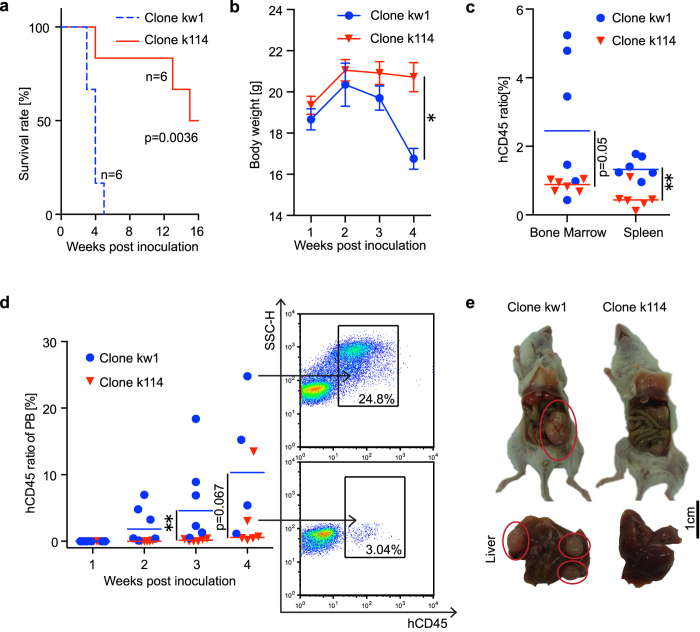
TALEN-mediated mono-allelic mutation of FLT3 reduces leukemia cell proliferation and improves the survival of NOD/SCID xenografted mice. (**a**) Kaplan‐Meier survival analysis of NOD/SCID mice inoculated with wild-type K562 cells (blue dotted line) or mutant K562 cells (red line). Each group includes 6 mice. The *log*-rank test was performed to determine statistical significance. (**b**) Weekly monitoring of the body weight of NOD/SCID mice inoculated with wild-type K562 (blue) or mutant K562 cells (red) for 4 weeks. The data are presented as the means ± SEM of the living mice at each time point. (**c**) The percentage of hCD45 + cells among the total cellular elements in the murine bone marrow and spleen at the endpoint of the experiment was determined *via* FACS analysis. The data are presented as scatter dot plots displaying the means (blue or red horizontal lines). (**d**) The left panel shows the serial monitoring of the percentage of hCD45 + cells in murine peripheral blood. The data are presented as scatter dot plots displaying the means (blue or red horizontal lines). Representative FACS results for the two groups are depicted in the right panel. The black arrows and boxes represent the gated hCD45 + cell populations. (**e**) Representative dissected tumors from mice sacrificed at 4 weeks post-inoculation from the two groups. The red circles indicated the visible tumor tissues located in the abdominal cavity (upper diagram) and on the murine liver (lower diagram) in the wild-type group. The unpaired t-test was performed to determine statistical significance. *P < 0.05; **P < 0.01.

The inhibitory effect of TALEN-mediated FLT3 haplo-insufficiency on K562 cell proliferation prompted us to further explore whether such effect is retained in a xenotransplantation model *in vivo*. We randomly selected a mutant K562 clone (clone k114) and a wild-type K562 clone (clone kw1) and intravenously injected 5 × 107 cells derived from either K562 clone into NOD/SCID mice. Both the wild-type and mutant K562 clones formed primary xenografts in the NOD/SCID mice, but the mutant clone displayed a significantly lower rate of morbidity than the wild-type clone (Fig. 4a). All 6 mice (100%) in the wild-type group were sacrificed within 5 weeks post-inoculation due to the first indication of xenografted leukemia (*e.g.*, weight loss, lethargy, or ruffled fur) or the elevation of the proportion of human CD45 + cells in the peripheral blood to 25%. Only 3 out of the 6 mice (50%) in the mutant FLT3 group exhibited signs of disease or peripheral blood infiltration within 16 weeks after inoculation. Loss of body weight began 2 weeks post-inoculation in the wild-type group, whereas no significant change in body weight was detected in the mutant group until 4 weeks after inoculation (Fig. 4b). At the end of the experiment, the presence of infiltrating leukemia cells in various organs was examined. On average, wild-type K562 cells accounted for more than 2% of the total cellular elements in the BM (Fig. 4c). In contrast, mutant K562 cells accounted for less than 1% of the total cellular elements in the BM. In good agreement with findings in the BM, significantly increased infiltration of hCD45 + cells in the murine spleen was detected in the wild-type group compared to the mutant group (Fig. 4c). The percentage of hCD45 + cells, which represents the leukemia cells, was serially monitored in peripheral blood (PB) after inoculation. Whereas the average percentage of engrafted wild-type K562 cells rapidly increased within the first 4 weeks post-inoculation, that of mutant K562 cells increased in a much slower manner (Fig. 4d). These results demonstrated that TALEN-mediated FLT3 haplo-insufficiency strongly retained its potent inhibitory effects on K562 cell proliferation *in vivo*. Indeed, in mice which happened to display signs of distress and to be sacrificed at the same time (4 weeks post-inoculation, 1 from the mutant group and 3 from the wild-type group), there were more visible tumor tissues of larger volume in the wild-type group than in the mutant group (Fig. 4e), although the statistical relevance of these data could not be evaluated due to the limited number of mice.

### TALEN-mediated FLT3 haplo-insufficiency induces multiple changes in the gene expression profile of K562 cells

**Figure 5:**
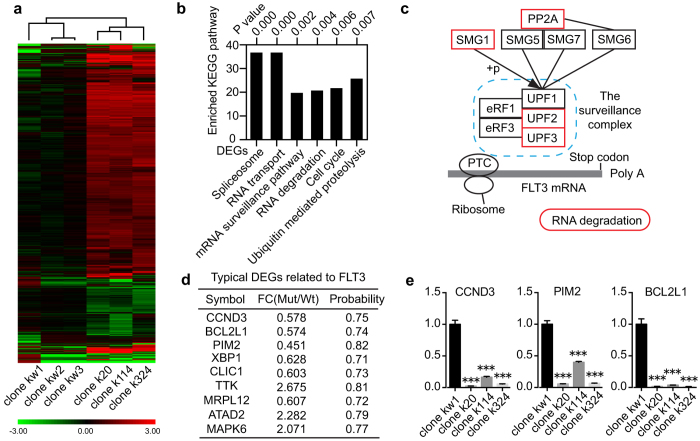
TALEN-mediated FLT3 haplo-insufficiency induces multiple changes in the gene expression profile. (**a**) RNA-*seq* and cluster analysis was performed on six isogenic clones. In the heatmap, each column represents the gene expression profile of one clone, and each row corresponds to one gene. The differences in gene expression are shown in different colors: Red indicates up-regulation, and green indicates down-regulation. The dendrogram above the heatmap demonstrates the similarity between the samples. The colored bar below the heatmap indicates the log2 ratio of gene expression. (**b**) Most of the enriched pathways identified by KEGG pathway analysis. Each column represents the number of DEGs between the mutant clones and the wild-type clones corresponding to the KEGG pathway indicated below the table. The nominal *p*-value is indicated above the column. (**c**) Schematic view of the assembly of the surveillance complex and the nonsense-mediated decay process based on KEGG pathway map03015. The blue-dotted box represents the surveillance complex, and the red boxes indicate the up-regulated genes and the KEGG pathway in mutant K562 clones carrying a premature termination codon in the FLT3 exon. (**d**) Representative DEGs related to FLT3 signaling as identified by RNA-*seq* analysis between the mutant group (Mut) and the wild-type group (Wt). The higher the probability of the DEG, the more significant the change in the expression level. FC, fold change. (**e**) The expression levels of three downstream FLT3 target genes, CCND3, PIM2 and BCL2 L1, were validated by RT-PCR analysis in three isogenic clones. The relative expression levels were normalized to GAPDH. The error bars represent the SEM. ***P < 0.001.

To identify the changes in gene expression caused by TALEN-mediated mono-allelic disruption of FLT3, we compared the global gene expression profile between three isogenic K562 mutant clones (clones k20, k112, k324) and three randomly selected wild-type clones (clones kw1, kw2, kw3) using RNA-*seq* (Supplementary Table S2). All mutant clones, along with their isogenic wild-type counterparts, were maintained in serial passage culture for 4 weeks prior to RNA-*seq* analysis. The cluster dendrogram demonstrated that the gene expression patterns were closely related to the FLT3 mutant status of all clones (Fig. 5a). The K562 clones displayed similar expression patterns among the same FLT3 mutant status (Fig. 5a). Given that all six subclones underwent the same genome-editing procedures, our data strongly suggest that the gene expression differences between the mutant and wild-type clones resulted from TALEN-mediated gene mutation rather than clonal deviation. Thus, this cellular model was suitable to explore the gene expression alterations specifically associated with mutated FLT3.

To gain mechanistic insight into the phenotypes generated by TALEN-mediated mono-allelic disruption of FLT3, we searched for genes that were expressed at a minimum threshold level and were either up or down-regulated by at least 1.5-fold in the same direction in all three mutant clones, resulting in 1410 DEGs. KEGG pathway analysis was performed on these DEGs, revealing that the most prominent pathways identified were related to RNA processing (Fig. 5b). For example, 4 genes (UPF2, UPF3, SMG1 and PPP2R5C which encodes a regulatory subunit of protein phosphatase 2A (PP2A)) which participate in the assembly of the mRNA surveillance complex^20^, along with 16 other genes (Supplementary Table S3) related to the RNA degradation pathway, were found to be substantially up-regulated in the FLT3 mono-allelic mutant group (Fig. 5c). To determine whether the activation of the mRNA surveillance pathway was cell line-specific or a common consequence of TALENs mediated mutations, we validated relative gene expression levels of UPF2, UPF3, SMG1 and PPP2R5C in three mutant OCI-AML3 clones and their isogenic wild-type controls. As shown in Supplementary Fig. S10, expression levels of UPF2, UPF3 and PPP2R5C were generally increased in clones o35, o166 and o253, whereas SMG1 expression was slightly decreased in those clones. These dramatic alterations in the gene expression profile of the mutant clones indicated activation of nonsense-mediated decay of FLT3 mRNA due to the TALEN-introduced premature termination codon, further supporting our previous findings of decreased FLT3 transcript levels in the FLT3 mutant clones (Fig. 2a).

DEG analysis further revealed that three well-known downstream target genes (CCND3, BCL2L1 and PIM2) of MAPK or JAK-STAT were down-regulated in the mutant group (Fig. 5d and Supplementary Table S4), which was validated by subsequent RT-PCR analysis (Fig. 5e), indicating down-regulated FLT3 signaling^21^,^22^,^23^. Moreover, in our RNA-*seq* data, we identified 7 DEGs (CCND3, XBP1, CLIC1, TTK, MRPL12, ATAD2 and MAPK6) which belong to a previously documented gene set associated with inhibited FLT3 signaling (Fig. 5d and Supplementary Table S4)^24^. Among those 7 DEGs, 6 genes were dysregulated in the same direction in our RNA-*seq* profile compared to previously reported microarray data, with only one exception (MAPK6) (Supplementary Table S5)^24^.

## Discussion

Identifying the critical molecular events caused by a given gene mutation is essential for designing effective targeted therapies. To achieve this goal, generally applicable and well-validated analytic tools play an important role and are in urgent need. Here, using a designed TALENs approach, we generated a panel of isogenic clones carrying a mono-allelic mutation of FLT3 in K562 and OCI-AML3 leukemia cells. We hypothesized that the resulting isogenic clones, which theoretically carried the same genetic background except for the FLT3 mutant status, serve as a cellular model to characterize the biological effects and critical molecular events caused by TALEN-mediated disruption of the FLT3 gene.

We have addressed several important issues to validate the TALEN-mediated cellular model. First, the present data showed that the genomic editing approach successfully disrupted the JM domain of FLT3 in a single allele while generating no detectable off-target effects on the genome. The introduced genetic profile produced a premature termination codon in FLT3 of a single allele, which was confirmed based on the reduced transcriptional levels of FLT3 and the substantially decreased autophosphorylation of FLT3 receptors in the mutant clones. More importantly, TALEN-mediated FLT3 haplo-insufficiency impaired leukemia cell proliferation capacity, and these potent inhibitory effects on K562 cell proliferation were retained *in vivo*. These findings strongly indicate that this genomic editing approach precisely mimics the effects of spontaneous gene mutations^25^,^26^. Second, the generation of mutant clones requires multiple experimental steps, and the extent to which the experimental procedures alter the phenotypes of selected clones must be addressed. We found that there was no significant difference in FLT3 transcription, signaling transduction or proliferation capacity either between the isogenic mutant clones or between the wild-type clones and the parental leukemia cell line, demonstrating that the experimental procedures had a negligible influence on the phenotypes of the selected clones. Third, the differences in the gene expression patterns between the FLT3 mutant and wild-type isogenic clones might only reflect the deviation among individual isogenic clones rather than the actual molecular events induced by the FLT3 mutation^27^. In our study, cluster dendrogram analysis of the RNA-*seq* data clearly demonstrated that the gene expression profile was exclusively determined by the FLT3 mutant status, indicating the reproducibility and reliability of the current strategy. Therefore, we concluded that this genome-editing approach serves as a robust and generally applicable platform to explore the molecular basis of a given gene mutation.

In the present study, we selected FLT3 for targeted disruption due to its well-known functions to promote cell proliferation. Previously, various approaches had been used to identify the key elements responsible for FLT3 signaling, including using small-molecule inhibitors and small RNA interference in cell lines^28^,^29^,^30^, establishing knock-in/knock-out transgenic mouse models^31^,^32^ and utilizing primary patient samples^33^. Despite the invaluable insight gained from these studies, to our knowledge, the present investigation is the first study to use isogenic cell-line based models generated using genome-editing technology to explore the critical molecular events underlying FLT3 haplo-insufficiency. Clearly, activation of nonsense-mediated decay in FLT3 mutant clones was caused by TALEN-introduced premature termination. Moreover, the critical molecular events associated with FLT3 haplo-insufficiency revealed by RNA-*seq* well corroborated with the results of previous studies. For instance, based on microarray analysis, Kim *et al.* identified a set of 35 genes consistently affected by FLT3 inhibition using small-molecule inhibitors in cell-lines harboring FLT3-ITD mutations. Among those 35 genes, 7 genes were identified to be DEGs between the isogenic mutant and wild-type clones in this study, 6 of which were dysregulated in the same manner when FLT3 signaling was inhibited.

Ideally, the optimal cell models for FLT3 function study should be cell lines with strong FLT3 wild-type expression, such as THP-1, or cell lines harboring FLT3-ITD mutations such as MV4-11 and MOLM-13. Unfortunately, our adopted transfection methods had resulted in unacceptable cell death, which might reflect the profound addiction of these cells to FLT3 signaling. Therefore, we chose K562 and OCI-AML3 cells that are less addicted to FLT3 signaling for survival. Moreover, their myelogenous leukemia origin can in part represent the abnormal signaling of acute leukemia. Indeed, by using sensitive analytic approaches such as immunoprecipitation and RNA-*seq*, we were able to dissect the molecular signaling changes in K562 isogenic clones. Interestingly, our data demonstrated that, even in FLT3 inhibitor-resistant K562 and OCI-AML3 cells, profound inhibition on cell proliferation could still be achieved providing FLT3 signaling is disrupted by more potent and sustained approaches such as TALENs. The present findings also highlight the therapeutic potential of TALENs for leukemia therapy by disrupting FLT3 signaling.

Notably, more than 50% of reduction in FLT3 expression was frequently observed in FLT3 haplo-insufficient clones despite the deviation between isogenic clones. Theoretically, for a cell line harboring two alleles of FLT3, a heterozygous mutation in FLT3 mediated by TALENs should result in approximately 50% of reduction of protein levels. A possible explanation for this deviation is due to allele-specific DNA methylation. Allelic asymmetries in DNA methylation has been found associated with allelic expression imbalance^34^. And several lines of evidence have suggested that TALENs have easier access to an endogenous genomic site being less methylated^35^,^36^,^37^. Therefore, TALENs might preferentially target and disrupt the relatively lightly methylated allele with stronger expression, causing more reduction in whole protein level.

This study provided evidence for the feasibility and reliability of isogenic cell line-based models established using TALENs technology to investigate the molecular bases of leukemia. Using carefully crafted homologous donors and advanced genome-editing tools, many recurrent somatic mutations (including point mutations, insertions, and even chromosomal translocations) associated with acute leukemia can be easily generated to establish various isogenic cellular models in the future^17^,^38^. These models will facilitate the extraction of the subtle molecular events caused by a single mutation from a complex genetic background and will provide novel insight into drug design for acute leukemia.

## Methods

### Construction and validation of customized TALEN expression vectors

TALENs targeting the FLT3 exon 14 locus were designed using the online software TALEN-NT^39^. The starting materials, including the nucleotide-recognizing TALE single unit vectors and the TALEN expression vectors, were kind gifts from Bo Zhang (Peking University). The TALEN expression vectors were constructed using the “Unit Assembly” method to contain the Sharkey-AS and Sharkey-RR forms of the FokI cleavage domains as described previously^40^.

The T7 endonuclease I assay was performed to validate and quantify the NHEJ-mediated mutations in the endogenous FLT3 gene as previously described^41^. Genomic DNA was isolated from K562 cells transfected with TALEN-encoding plasmids or an empty expression vector using the DNeasy Blood and Tissue Kit (Qiagen) according to the manufacturer’s instructions. The region of DNA containing the TALEN target site was PCR-amplified using a forward primer (5′-TCTGTTTCATCGCTGAGTGAC-3′) and a reverse primer (5′-TTTCCAAAAGCACCTGATCC-3′) in which the fluorescent tag FAM was labeled at each 5′ end. The amplicons were purified using the GeneJET PCR Purification Kit (Thermo Scientific), and 200 ng of purified PCR product was denatured and re-annealed in NEBuffer 2 (New England Biolabs) using a thermocycler according to the following protocol: 95 °C, 5 min; 95–85 °C at −2 °C/s; 85–25 °C at −0.1 °C/s; and hold at 4 °C^42^. The hybridized PCR products were treated with 10 U of T7 endonuclease I at 37 °C for 15 min in a reaction volume of 20 μl. The reactions were stopped by adding 2 μl of 0.5 M EDTA, followed by analysis *via* capillary electrophoresis using an ABI3750 sequencer. The sum of the area under the TALEN-specific cleavage peaks (expressed as the percentage of the parent amplicon peak, denoted fraction cleaved) was used to estimate the gene modification levels according to the following previously described equation: % gene modification = 100 × (1-(1-fraction cleaved)^1/2^)^42^.

### Cell culture, transfection and clone screening

The cell lines K562, MOLM-13, THP-1 and Jurkat were purchased from American Type Culture Collection and were maintained in RPMI 1640 supplemented with 10% fetal bovine serum (FBS) (Gibco). The OCI-AML3 cell line was purchased from Leibniz Institute DSMZ-German Collection of Microorganisms and Cell Cultures and was cultured in alpha-MEM supplemented with 10% FBS. For transient transfection, the TALEN expression plasmids (6 μg per TALEN) were nucleo-transfected into 1 × 10^6^ K562, OCI-AML3 or Jurkat cells using Amaxa nucleofector II and solution V (Lonza) applying program T16, solution T (Lonza) applying program X01 or solution V applying program X01, respectively. After incubation at 30 °C for 48 hours following transfection^43^, the cells were seeded in 96-well plates at limited dilution (average of 0.4 cells per well). Each clone was cultured for two to three weeks, and a portion of the cells in each well was collected for PCR analysis to detect NHEJ-mediated insertion or deletion using the same primers described in “Construction and validation of customized TALEN expression vectors”. The fragments for PCR amplification were subcloned into the pEASY-T1 Simple Cloning Vector (TransGen Biotech, China) to confirm mutation of a single allele. The two working parental cell lines, K562 and OCI-AML3, along with three clones (clone k324, clone o253 and clone KI-H6) were sent to China Center for Type Culture Collection (CCTCC, Wuhan University, Wuhan 430072, China) for cell line identification (Supplementary Fig. S11–15).

### Off-target analysis

The TALE-NT 2.0 Paired Target Finder was employed to identify potential off-target sites in silico for our designed TALENs using the following recommended criteria: spacer length, 12–30 base pairs; threshold score, 3.0^39^. The 20 most plausible candidates other than the on-target site were PCR-amplified and sequenced using the primers described in the Supplementary Table S6.

### Real-time reverse transcription-polymerase chain reaction (RT-PCR)

Total RNA was isolated using the RNeasy kit according to the manufacturer’s instructions. RNA quantification was performed using a NanoDrop micro-volume spectrophotometer (Thermo Fisher), and the mRNA integrity was verified via agarose gel electrophoresis. Then, RT-PCR was performed using 2 μg of total RNA and random primers. Quantitative RT-PCR was performed using a CFX96 Touch^TM^ Real-Time PCR Detection System (Bio-Rad) according to the following thermocycler program for each locus: 5 min of pre-incubation at 95 °C followed by 40 cycles of 15 s at 95 °C, 15 s at 60 °C, and 30 s at 72 °C. All primers used are listed in Supplementary Table S7. Data analysis, including the fold-change in gene expression, was performed using the same amount of total RNA.

### Immunoprecipitation and Western blot

Immunoprecipitation and immunoblotting were performed as previously described^28^,^44^,^45^. The cells were washed twice with PBS and starved overnight in medium containing 0.5% serum. Then, the cells were stimulated for 5 minutes at 37 °C using 40 ng/ml FLT3 ligand (Peprotech). Subsequently, the cells were washed once with ice-cold PBS and incubated in lysis buffer (Beyotime, China). After incubation on ice for 10 minutes, the cell lysates were clarified at 20,000 g for 10 minutes at 4 °C. For immunoprecipitation, the cell lysates were incubated in either control rabbit polyclonal serum or an antibody against FLT3 (Sc-480; Santa Cruz Biotechnology) overnight; then, Protein A/G-Plus-Sepharose (Santa Cruz Biotechnology) was added and incubated for an additional 2 hours. The immunoprecipitates were washed 4 times with lysis buffer, resolved *via* sodium dodecyl sulfate–polyacrylamide gel electrophoresis (SDS-PAGE), transferred to Immobilon P membranes (Millipore, Bedford, MA), and probed overnight at 4 °C using either 4G10 (a phosphotyrosine-specific antibody) to detect p- FLT3 (phosphorylated FLT3) or an antibody against FLT3 (Sc-479; Santa Cruz Biotechnology) to detect total FLT3. Immunoblotting analysis was performed using Enhanced Chemiluminescence (ECL) Western Blotting Detection reagents (Pierce). For detection of p- Flt3, Flt3, p–signal transducer and activator of transcription 5 (Stat5), Stat5, p–Erk1/2, t-Erk1/2, t-Erk1, p-Akt, Akt, p-Abl and Bcr-Abl, standard immunoblotting procedures were performed using anti–Flt3 (Sc-479; Santa Cruz Biotechnology), anti–p-Flt3, anti–p-Stat5, anti–p-Akt, anti–Akt, anti–p-c-Abl (Cell Signaling Technology), anti–p-Erk1/2, anti–Erk1/2, anti–Erk1 (Abcam), anti–Stat5 and anti-Bcr antibodies (Santa Cruz Biotechnology). The total blot images of the Western blots were provided as Supplementary Fig. S16 and Supplementary Fig. S17.

### Cell proliferation and colony-forming assay

Cell Counting Kit-8 (Beyotime, China) was used to determine the cell viability. The cells were seeded in 96-well plates at 5000 cells per well and incubated for 24, 48, 72 or 96 h. At the end of incubation, the cell proliferation reagent WST-8 (10 μl) was added to each well, which was incubated for 4 h at 37 °C. The number of viable cells was estimated based on measurement of the optical density (OD) at 450 nm. Alternatively, the cells were stained with carboxyfluorescein succinimidyl ester (CFSE) and cultured at 1 ×10^6^/well. Cell proliferation was determined *via* flow cytometry (FACS Calibur, CA, USA) on day 3 and day 4 using ModFit software. For the colony-forming assay, 1 ml of a culture mixture containing RPMI-1640 (for K562 or Jurkat) or alpha-MEM (for OCI-AML3), 1% methylcellulose, and 10% FBS was seeded in a 24-well plate. Each parental cell line and its isogenic clonal populations were seeded at a concentration of 300 cells/well for K562, 500 cells/well for Jurkat, or 1000 cells/well for OCI-AML3. The assays were performed in triplicate. The cells were cultured at 37 °C in humidified air containing 5% CO2 for 10 days. The colonies containing more than 40 cells were counted under a microscope on day 7.

### Transplantation of isogenic K562 clones into NOD/SCID mice

All mouse studies were conducted in accordance with the recommendations in the Guide for Care and Use of Laboratory Animals of the National Institutes of Health and the guidelines of the Institutional Committee of Animal Care and Treatment at Tongji Hospital, and were approved by the Ethics Committee of Tongji Hospital. NOD/SCID mice were housed under sterile conditions using HEPA-filtered microisolators and were fed irradiated food and acidified water in the animal facility of Crown Bioscience, Inc. (Beijing). Adult mice (6–8 weeks old) were sublethally irradiated with 200 cGy to the total body 24 h before injection of K562 cells. The K562 cells were washed twice in PBS, cleared of aggregates and debris using a 0.2 mm cell filter, and suspended in PBS at a final concentration of 5 million cells per 200 ml of PBS per mouse for intravenous injection *via* the tail vein. Daily monitoring of mice for symptoms of disease (ruffled coat, hunched back, weakness and reduced motility) determined the time for sacrifice of the injected animals displaying signs of distress. If no signs of distress were detected, the mice were analyzed 16 weeks after injection. Peripheral blood (PB) cells were collected from the xenotransplanted NOD/SCID mice each week after transplantation. To detect K562 cells in the mouse PB, the cells were stained with mouse anti-human CD45 (BD 555485) and analyzed *via* flow cytometry (FACS Calibur, CA, USA). For analysis of the sacrificed mice, mixed bone marrow (BM) from tibias and femurs, the spleen, the liver and the kidney were dissected in a sterile environment, flushed in PBS and prepared as single cell suspensions for analysis of the percentage of hCD45 + cells *via* flow cytometry (FACS Calibur, CA, USA). Successful engraftment was defined as the presence of at least 0.1% human CD45 positive (hCD45 + ) cells in the mouse BM.

### RNA-*seq* and differential gene expression analysis

Total cellular RNA was extracted from the cell populations of three randomly selected wild-type K562 clones (clones kw1, kw2 and kw3) and three isogenic mutant clones (clones k20, k114 and k324) using TRIzol reagent (Invitrogen) according to the manufacturer’s protocol. The quality and quantity of the RNA samples were examined using an ND-2000 Spectrophotometer (Nano-Drop) and *via* denaturing agarose gel electrophoresis. All RNA samples were treated with DNase-I for later use. RNA library preparation and sequencing using Illumina HiSeq^TM^ 2000 were conducted at Beijing Genomics Institute (BGI, Shenzhen). The high-quality reads were aligned to the human reference genome (NCBI Build 36.1) using SOAPaligner-v2.21 software (BGI). The matched reads were aligned to Human Refseq mRNA (NCBI). The sequences that aligned with an individual transcript were counted digitally. The expression levels for each gene were normalized to the reads per kilobase of the target exon per million mapped reads (RPKM) to facilitate the comparison of transcripts between samples.

The “K-means” of the gene expression levels of the three wild-type clones were used as a global background for all six samples. The differentially expressed genes (DEGs) between each sample and the background were identified using a rigorous algorithm developed by BGI using the threshold of “FDR ≤ 0.001 and the absolute value of log2Ratio ≥ 1”^46^. Expression pattern analysis of the DEGs was performed using Cluster^47^ software and Java Treeview^48^ software.

The NOIseq method was applied to screen the DEGs between the wild-type and mutant group using the filtering conditions “fold change ≥ 1.5 and probability ≥ 0.7”^49^. KEGG pathway enrichment analysis was performed by annotating the DEGs to the KEGG database using BLAST and Blast2GO according to the hypergeometric distribution model.

Raw data files and processed expression files have been deposited in NCBI’s Gene Expression Omnibus and are accessible through GEO Series accession number GSE69678 (http://www.ncbi.nlm.nih.gov/geo/query/acc.cgi? acc =GSE69678).

### Statistical analyses

All of the statistical analyses were performed using GraphPad Prism V6.0 (GraphPad Software, La Jolla California USA). The significance of the differences between selected groups was evaluated via one-way analysis of variance followed by Dunnett’s multiple comparisons test unless stated otherwise.

## Additional Information

**How to cite this article**: Wang, J. *et al.* TALENs-mediated gene disruption of FLT3 in leukemia cells: Using genome-editing approach for exploring the molecular basis of gene abnormality. *Sci. Rep.* **5**, 18454; doi: 10.1038/srep18454 (2015).

## Supplementary Material

Supplementary Information

## Figures and Tables

**Table 1:** Summary of the off-target analysis to isogenic clones.

Clone analyzed	Putative off-target sites examined	Mutated sites detected (reference sequence)	Comparison to parental K562 sequence
Clone kw1	20	2	Identical (20/20)
Clone k20	20	2	Identical (20/20)
Clone k114	20	2	Identical (20/20)
Clone k324	20	2	Identical (20/20)

The 20 most possible off-target sites identified using the TALE-NT 2.0 Paired Target Finder were sequenced and aligned with either reference sequence (human reference genome, NCBI Build 36.1) or parental K562 sequence.
