# Supplementary material for: TALENs-mediated gene disruption of FLT3 in leukemia cells: Using genome-editing approach for exploring the molecular basis of gene abnormality
Source: Sci Rep. 2015 Dec 16;5:18454. doi: 10.1038/srep18454 (PMC4680874; doi:10.1038/srep18454)
Supplement: Supplementary Information [file srep18454-s1.pdf]

Supplementary information

TALENs-mediated gene disruption of FLT3 in leukemia cells: Using genome-editing approach for exploring the molecular basis of gene abnormality

*Jue Wang[1,#], Tongjuan Li[1,#], Mi Zhou[1], Zheng Hu[2], Xiaoxi Zhou[1], Shiqiu Zhou[1], Na Wang[1], Liang Huang[1], Lei Zhao[1], Yang Cao[1], Min Xiao[1], Ding Ma[2], Pengfei Zhou [3], Zhen Shang[1, *] and Jianfeng Zhou[1,2, *]*

1 Department of hematology, Tongji Hospital, Tongji Medical College, Huazhong University of Science and Technology, Wuhan, Hubei, China; 2 Cancer Biology Research Center, Tongji Hospital, Tongji Medical College, Huazhong University of Science and Technology, Wuhan, Hubei, China; 3 Wuhan YZY Bio-Pharma Co., Ltd., Wuhan, Hubei, China

[#] Note: Jue Wang and Tongjuan Li contributed equally to this work.

* Corresponding authors: Dr. Jianfeng Zhou, Tel and Fax: 86 (27) 83662680, E-mail: *jfzhou@tjh.tjmu.edu.cn*; Dr. Zhen Shang, Tel and Fax: 86 (27) 83662681, E-mail: *shangzhen1006@aliyun.com*; Address: No.1095 Jie Fang Avenue, Hankou, Wuhan 430030, P.R. China.

Supplementary Figure S1. Representative flow cytometry results of nucleofection efficiency for K562 cells.

The upper panel indicates null green fluorescent protein (GFP) expression in K562 cells after incubation with 2ug pmaxGFP vectors (Lonza) for 48 hours as negative control. The lower panel shows that over 90% transfection efficiency was achieved under the experimental conditions described in METHODS.

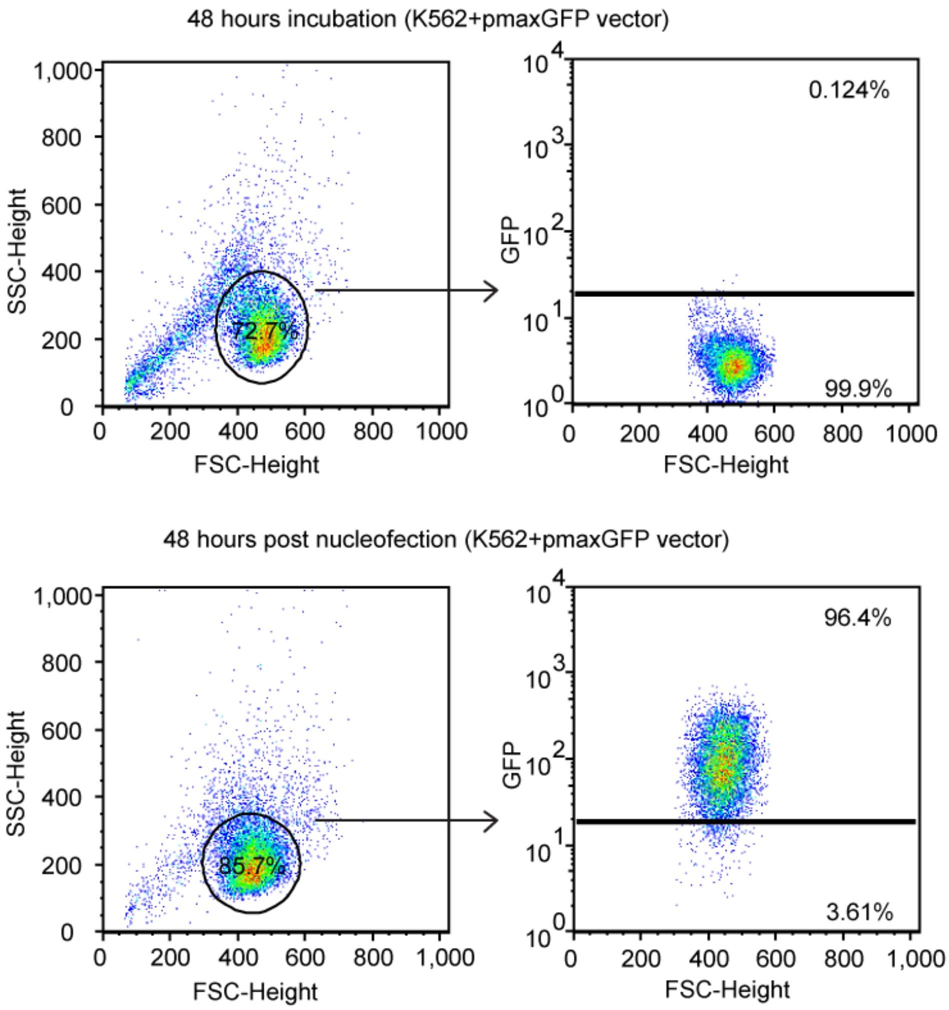

Supplementary Figure S2. Representative image of immunoblot analysis of basal FLT3 expression level in a panel of working leukemia cell lines.

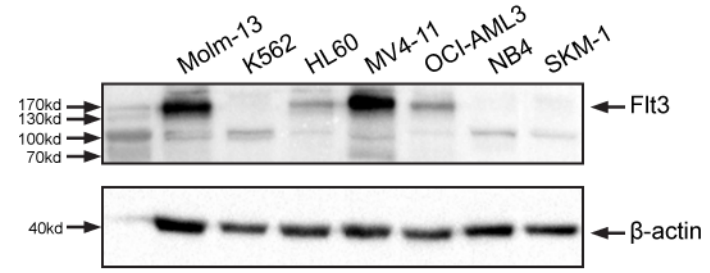

Supplementary Figure S3. Immunoprecipitation and Western blot of FLT3 in Jurkat, THP-1 and K562 cells. Displayed is the same SDS-PAGE gel under different exposure time. The total input of immunoprecipitates or cell lysate were denoted. The FLT3 bands were indicated by black arrows.

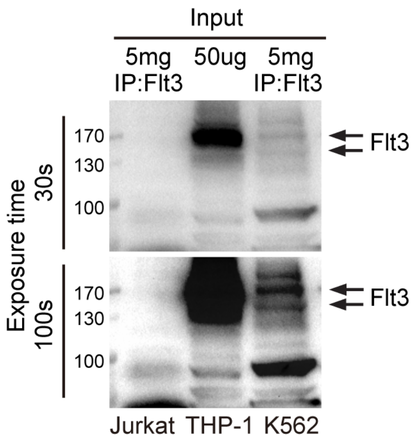

Supplementary Figure S4. Identification of FLT3 peptides in K562 cells by mass spectrometry.

(a) Displayed is the identification of FLT3 208-215 peptides EESPAVVK from wild-type K562 sample. MS2 fragmentation spectrum of the precursor ion is shown. A series of b and y product ions generated by HCD fragmentation were detectable. (b) Identification of FLT3 827-834 peptides ICDFGLAR by LC-MS/MS as in (a).

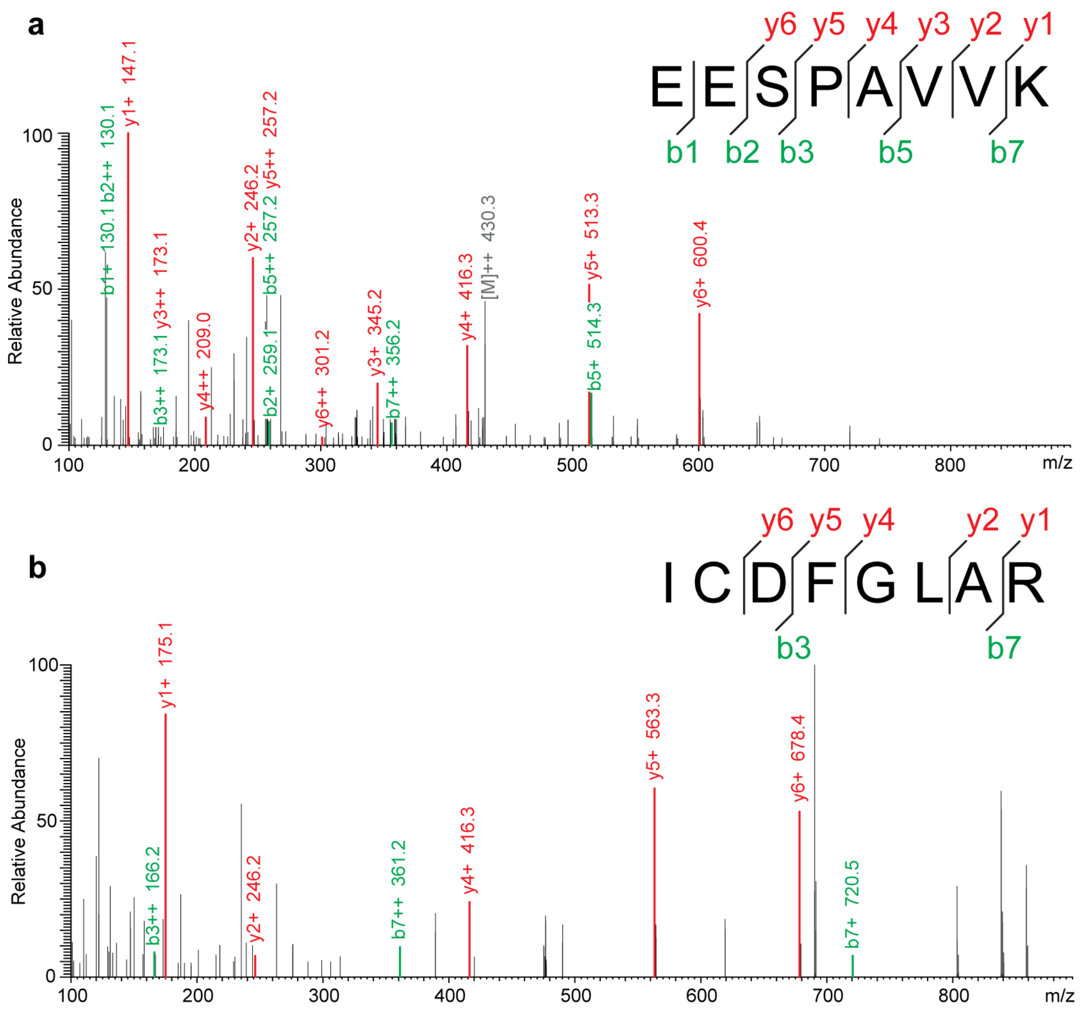

Supplementary Figure S5. Single-allelic disruption of FLT3 does not alter FLT3 signaling in Jurkat cells. (a) Representative image of immunoblot analysis of the FLT3 autophosphorylation levels in parental Jurkat cells, isogenic wild-type Jurkat clone jw1 and two mutant clones (j105 and j212) with single-allelic disruption of genomic FLT3. THP-1 was used as positive control. (b) Representative image of immunoblot analysis of the signaling mediators downstream of FLT3 in Jurkat. The cells were pre-starved of growth factors for 12 h and were subsequently exposed to the FLT3 ligand for 10 min before Western blot analysis.

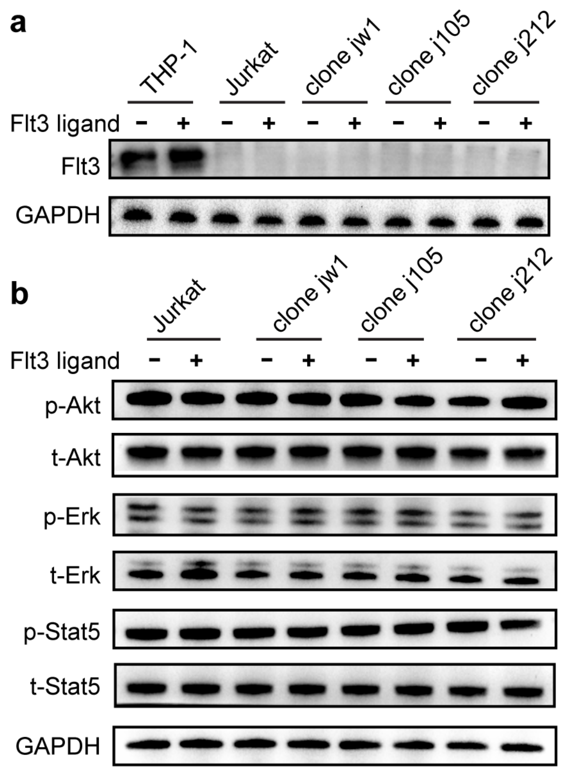

Supplementary Figure S6. FLT3 siRNA knock-down in OCI-AML3 and THP-1 and K562 resulted in similar FLT3 signaling changes to TALENs mediated haploinsufficiency. (a) RT-PCR analysis of FLT3 expression 48 hours after siRNA transfection. (b) SiRNA induced FLT3 and downstream signaling alterations were assessed in cells depicted by immunoprecipitation and immunoblot. For K562 cells, immunoblotting was performed following immunoprecipitation with FLT3 antibody. For THP-1 and OCI-AML3 cells, standard

immunoblotting analysis was conducted. IP, immunoprecipitation; NS, non-specific control siRNA.

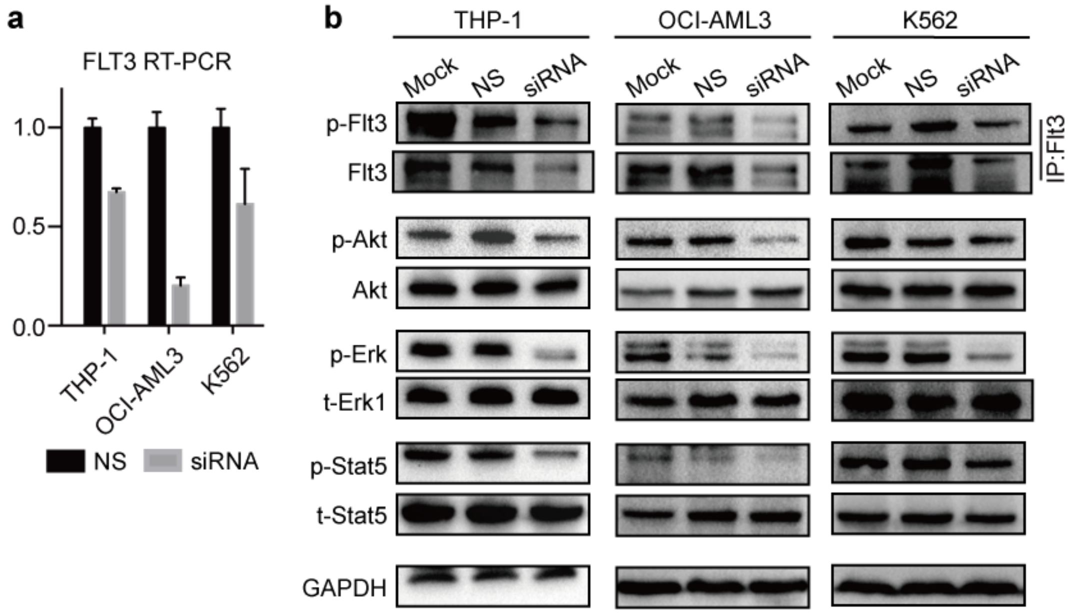

Supplementary Figure S7. TALENs induced FLT3 disruption in Jurkat cells does not influence proliferation and colony-forming capacity. (a) Growth curves of the parental cell lines and its isogenic clones were detected *via* the CCK-8 assay. All OD values were expressed as the fold changes compared to those of the 0 h time point. (b) The colony-forming assay in methylcellulose complete medium. Quantitative analyses of the colony numbers are shown. For statistical significance, the values were expressed as the means ± SEM of three independent experiments. ns, not significant.

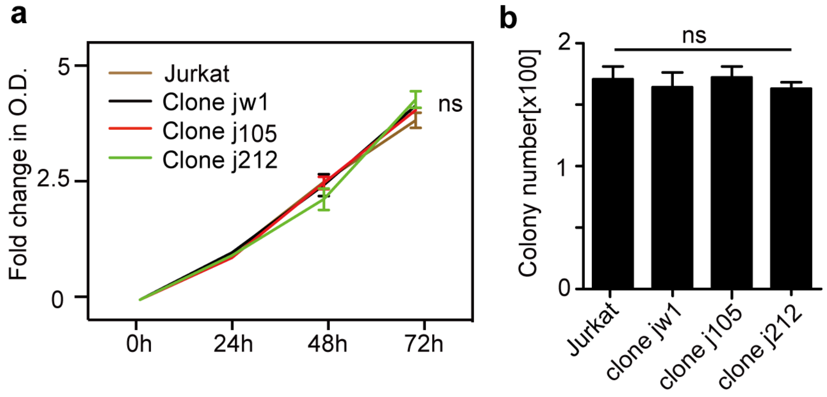

Supplementary Figure S8. Growth comparison between isogenic K562 clones with different FLT3 mutant status. (a) Sanger sequencing results of a heterozygous K562 clone (KI-H6) harboring an artificial FLT3-ITD mutation in single allele. (b) CCK-8 assay was performed to compare growth capacities of three isogenic K562 clones (kw1, KI-H6 and k114) with different FLT3 mutant status. For statistical significance, the values were expressed as the means ± SEM of three independent experiments. ns, not significant; **, $P < 0.01$.

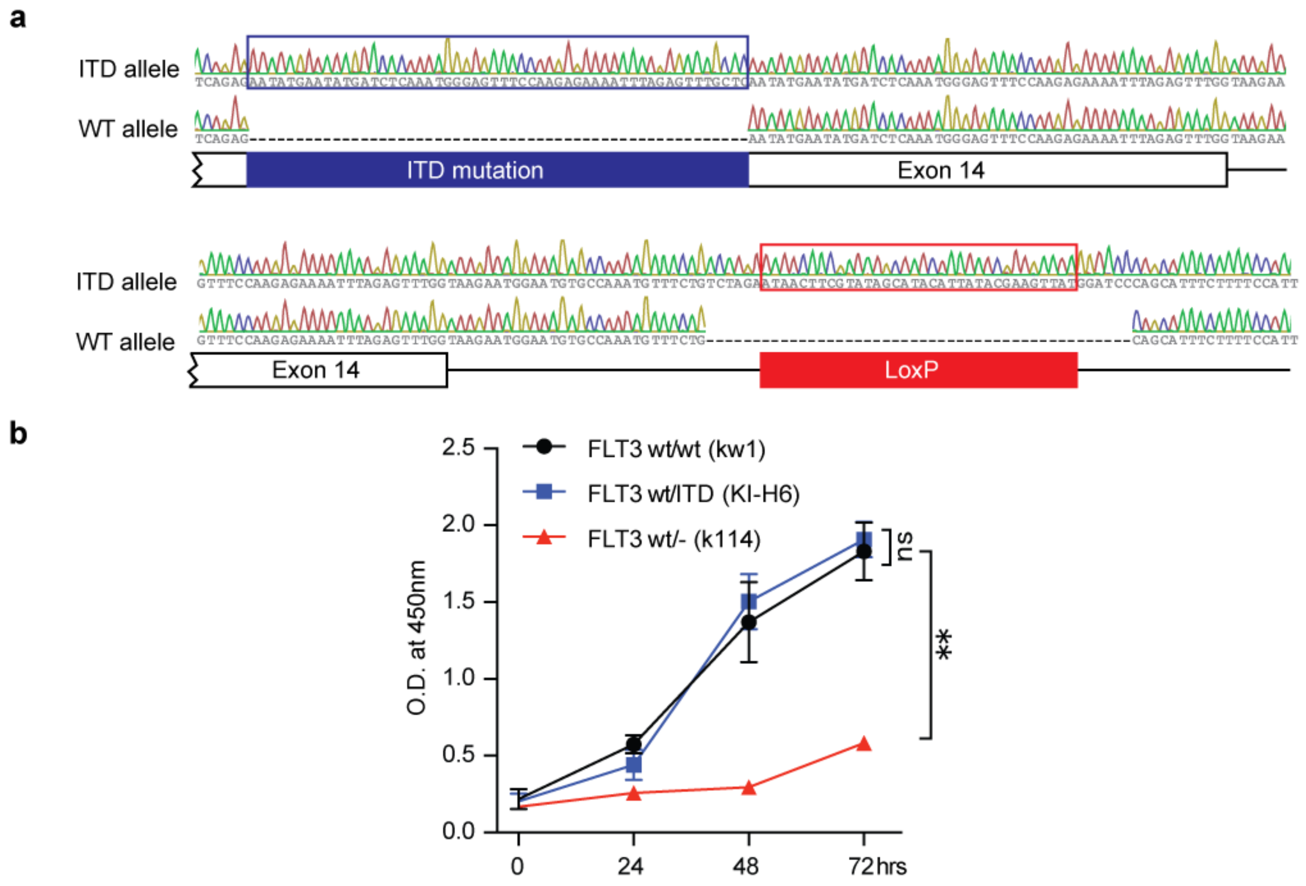

Supplementary Figure S9. Impacts of tyrosine kinase inhibitors (TKI), Sorafenib and Quizartinib (AC220), on cell proliferation of different leukemia cell lines. Growth inhibition of cells treated with various concentrations of TKIs for 72 hours were detected *via* the CCK-8 assay. All OD values were expressed as the percentage comparing with the OD in the control group. The values were expressed as the means ± SEM of three independent experiments.

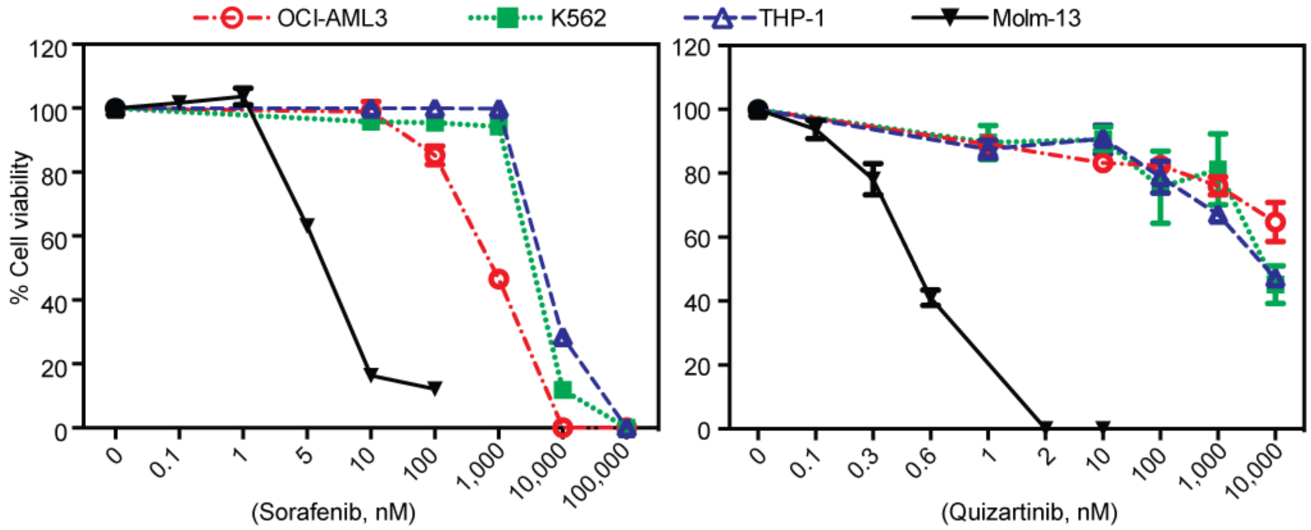

Supplementary Figure S10. Quantitative RT-PCR analysis of UPF2, UPF3, PPP2R5C and SMG1 expression in OCI-AML3 and its isogenic clones. The relative expression levels normalized to GAPDH are expressed as the means ± SEM. NS, not significant; *, $P < 0.05$; **, $P < 0.01$ compared to those of the parental cells.

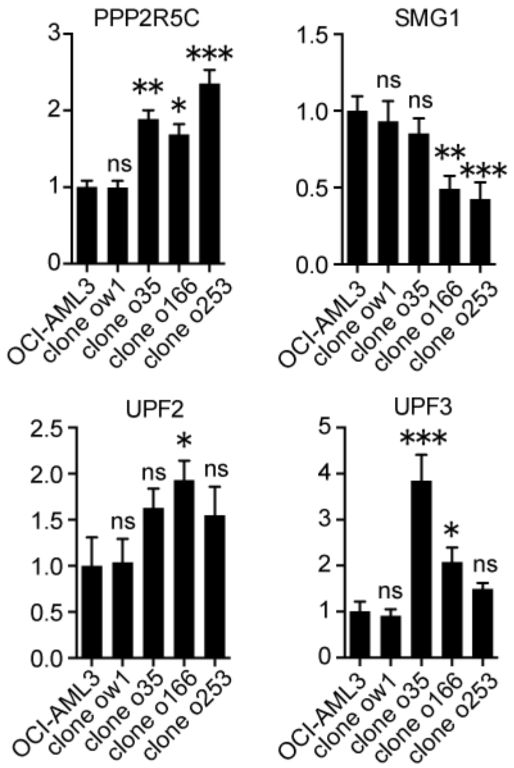

Supplementary Figure S11. Cell line identification of working parental K562 confirmed by China Center for Type Culture Collection (CCTCC, Wuhan University, Wuhan 430072, China).

中国典型培养物保藏中心

CHINA CENTER FOR TYPE CULTURE COLLECTION (CCTCC)

Wuhan University, Wuhan 430072, China

Phone: 86-027-68752093 **Fax:** 86-027-68754833 **Email:** shenchao@whu.edu.cn

12-15-2014

Entrusted by Laboratory of Tongji Hospital of Tongji Medical College, Huazhong University of Science and Technology，CCTCC has conducted identification experiments on the K562 cell line, and come to the following conclusions:

1. There was no third allele found in all the locations of K562 cell line, it indicating that there was no cross-contaminant of human source cell line.
2. Compared the STR data of cell line in the databases of ATCC DSMZ and JCRB, all the locations of K562 were exactly matched with the locations of K-562 cells found in all the three cell banks, so it is K562 cell line (Table 1).

Director's Signature：

China Center for Type Culture Collection

Table 1. The alleles of 21 locations in K562 cell line

K562 cell line（Fig. No.XB2553）		
Marker	Allele1	Allele 2
D19S433	14	14.2
D5S818	11	12
D21S11	29	31
D18S51	15	16
D6S1043	11	11
AMEL	X	X
D3S1358	16	16
D13S317	8	8
D7S820	9	11
D16S539	11	12
CSF1PO	9	10
Penta D	9	13
D2S441	10	14
vWA	16	16
D8S1179	12	12
TPOX	8	9
Penta E	5	14
TH01	9.3	9.3
D12S391	23	23
D2S1338	17	17
FGA	21	24

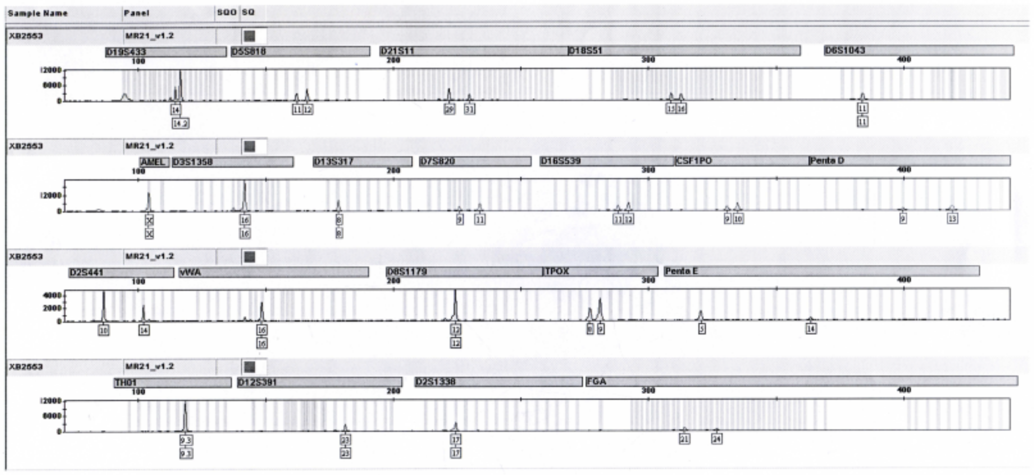

Supplementary Figure S12. Cell line identification of working parental OCI-AML3 confirmed by China Center for Type Culture Collection (CCTCC, Wuhan University, Wuhan 430072, China).

中国典型培养物保藏中心

CHINA CENTER FOR TYPE CULTURE COLLECTION (CCTCC)

Wuhan University, Wuhan 430072, China

Phone: 86-027-68752093 **Fax:** 86-027-68754833 **Email:** shenchao@whu.edu.cn

12-15-2014

Entrusted by Laboratory of Tongji Hospital of Tongji Medical College, Huazhong University of Science and Technology, CCTCC has conducted identification experiments on the OCI-AML3 cell line, and come to the following conclusions:

1. There was no third allele found in all the locations of OCI-AML3 cell line, it indicating that there was no cross-contaminant of human source cell line.
2. Compared the STR data of cell line in the databases of ATCC DSMZ and JCRB, all the locations of OCI-AML3 were exactly matched with the locations of OCI-AML3 cells found in the DSMZ cell bank, so it is OCI-AML3 cell line (Table 1).

Director's Signature:

China Center for Type Culture Collection

Table 1. The alleles of 21 locations in OCI-AML3 cell line

OCI-AML3 cell line (Fig. No.XB2554)		
Marker	Allele1	Allele 2
D19S433	15	17
D5S818	11	13
D21S11	29	30
D18S51	12	18
D6S1043	11	13
AMEL	X	X
D3S1358	16	16
D13S317	8	13
D7S820	9	10
D16S539	11	13
CSF1PO	11	12
Penta D	12	13
D2S441	14	14
vWA	17	18
D8S1179	10	12
TPOX	8	11
Penta E	11	13
TH01	8	10
D12S391	20	21
D2S1338	17	21
FGA	22	22

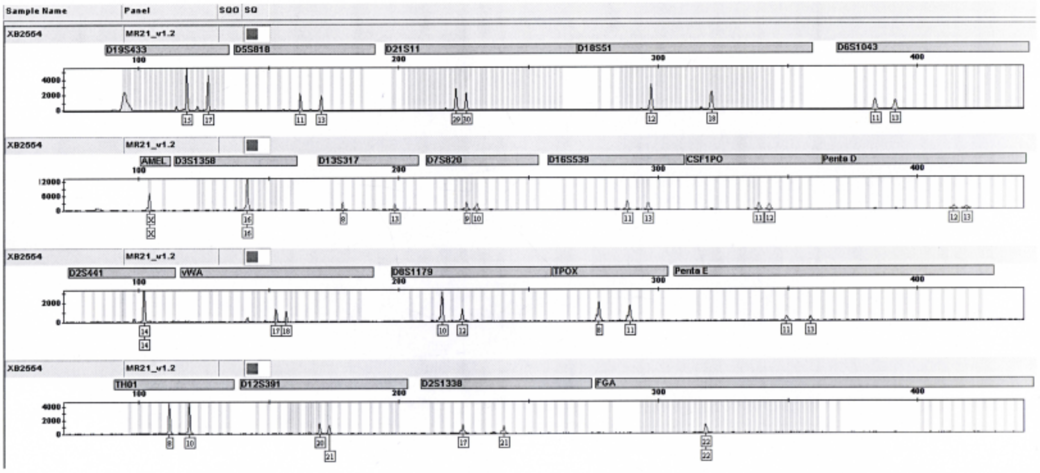

Supplementary Figure S13. Cell line identification of clone k324 (K562) confirmed by China Center for Type Culture Collection (CCTCC, Wuhan University, Wuhan 430072, China).

中国典型培养物保藏中心

CHINA CENTER FOR TYPE CULTURE COLLECTION (CCTCC)

Wuhan University, Wuhan 430072, China

Phone: 86-027-68752093 Fax: 86-027-68754833 Email: shenchao@whu.edu.cn

06-08-2014

Entrusted by Laboratory of Tongji Hospital of Tongji Medical College, Huazhong University of Science and Technology, CCTCC has conducted identification experiments on the K562(K324) cell line, and come to the following conclusions:

1. There was no third allele found in all the locations of K562(K324) cell line, it indicating that there was no cross-contaminant of human source cell line.
2. Compared the STR data of cell line in the databases of ATCC DSMZ and JCRB, all the locations of K562(K324) were exactly matched with the locations of K-562 cells found in all the three cell banks, so it is K-562 cell line (Table 1).

Director's Signature:

China Center for Type Culture Collection

Table 1. The alleles of 21 locations in K562(K324) cell line

K562(K324) cell line（Fig. No.XB3071）		
Marker	Allele1	Allele 2
D19S433	14	14.2
D5S818	11	12
D21S11	29	31
D18S51	15	16
D6S1043	11	11
AMEL	X	X
D3S1358	16	16
D13S317	8	8
D7S820	9	11
D16S539	11	12
CSF1PO	9	10
Penta D	9	13
D2S441	10	14
vWA	16	16
D8S1179	12	12
TPOX	8	9
Penta E	5	14
TH01	9.3	9.3
D12S391	23	23
D2S1338	17	17
FGA	21	24

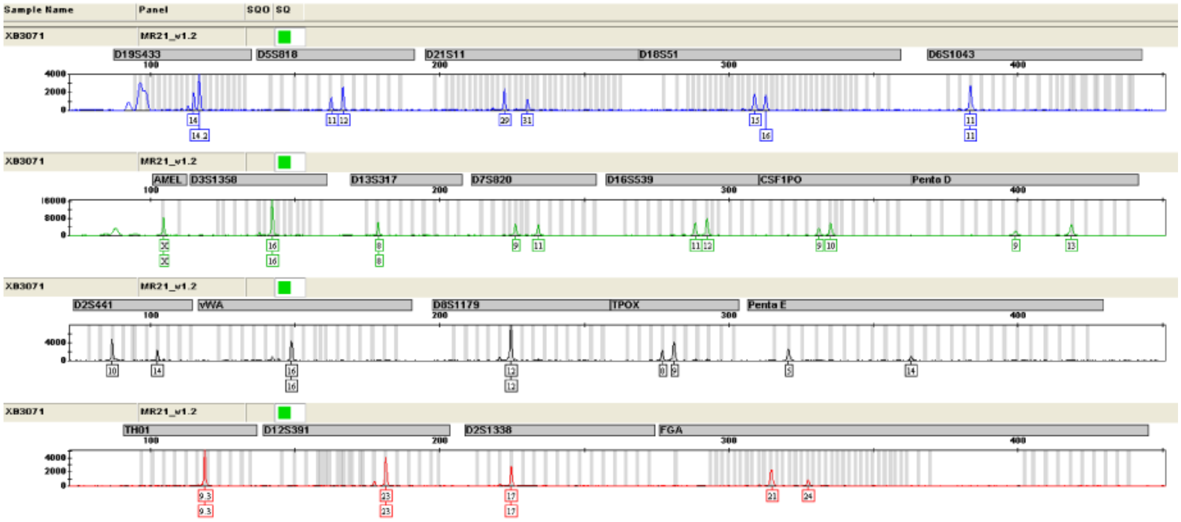

Supplementary Figure S14. Cell line identification of clone KI-H6 (K562) confirmed by China Center for Type Culture Collection (CCTCC, Wuhan University, Wuhan 430072, China).

中国典型培养物保藏中心

CHINA CENTER FOR TYPE CULTURE COLLECTION (CCTCC)

Wuhan University, Wuhan 430072, China

Phone: 86-027-68752093 **Fax:** 86-027-68754833 **Email:** shenchao@whu.edu.cn

06-08-2014

Entrusted by Laboratory of Tongji Hospital of Tongji Medical College, Huazhong University of Science and Technology，CCTCC has conducted identification experiments on the K562(KI-H6) cell line, and come to the following conclusions:

1. There was no third allele found in all the locations of K562(KI-H6) cell line, it indicating that there was no cross-contaminant of human source cell line.
2. Compared the STR data of cell line in the databases of ATCC DSMZ and JCRB, all the locations of K562(KI-H6) were exactly matched with the locations of K-562 cells found in all the three cell banks, so it is K-562 cell line (Table 1).

Director's Signature：

China Center for Type Culture Collection

Table 1. The alleles of 21 locations in K562(KI-H6) cell line

K562(KI-H6) cell line（Fig. No.XB3070）		
Marker	Allele1	Allele 2
D19S433	14	14.2
D5S818	11	12
D21S11	29	31
D18S51	15	16
D6S1043	11	11
AMEL	X	X
D3S1358	16	16
D13S317	8	8
D7S820	9	11
D16S539	11	12
CSF1PO	9	10
Penta D	9	13
D2S441	10	14
vWA	16	16
D8S1179	12	12
TPOX	8	9
Penta E	5	14
TH01	9.3	9.3
D12S391	23	23
D2S1338	17	17
FGA	21	24

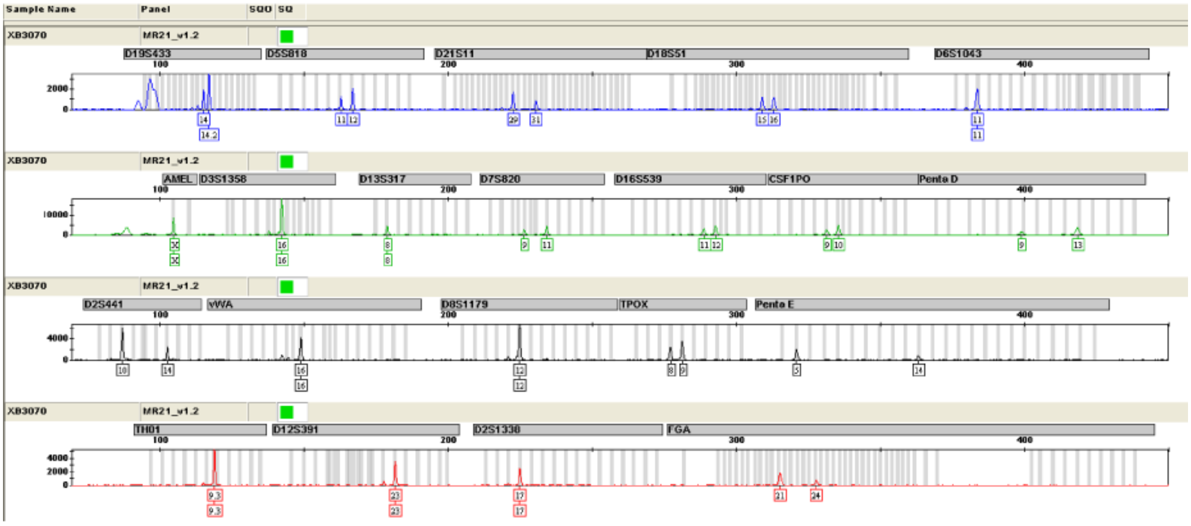

Supplementary Figure S15. Cell line identification of clone o253 (OCI-AML3) confirmed by China Center for Type Culture Collection (CCTCC, Wuhan University, Wuhan 430072, China).

中国典型培养物保藏中心

CHINA CENTER FOR TYPE CULTURE COLLECTION (CCTCC)

Wuhan University, Wuhan 430072, China

Phone: 86-027-68752093 Fax: 86-027-68754833 Email: shenchao@whu.edu.cn

06-08-2014

Entrusted by Laboratory of Tongji Hospital of Tongji Medical College, Huazhong University of Science and Technology, CCTCC has conducted identification experiments on the OCI-AML3(O253) cell line, and come to the following conclusions:

1. There was no third allele found in all the locations of OCI-AML3(O253) cell line, it indicating that there was no cross-contaminant of human source cell line.
2. Compared the STR data of cell line in the databases of ATCC DSMZ and JCRB, all the locations of OCI-AML3(O253) were exactly matched with the locations of OCI-AML3 cells found in DSMZ cell bank, so it is OCI-AML3 cell line (Table 1).

Director's Signature:

China Center for Type Culture Collection

Table 1. The alleles of 21 locations in OCI-AML3(O253) cell line

OCI-AML3(O253) cell line（Fig. No.XB3072）		
Marker	Allele1	Allele 2
D19S433	15	17
D5S818	11	13
D21S11	29	30
D18S51	12	18
D6S1043	11	13
AMEL	X	X
D3S1358	16	16
D13S317	8	13
D7S820	9	10
D16S539	11	13
CSF1PO	11	12
Penta D	12	13
D2S441	14	14
vWA	17	18
D8S1179	10	12
TPOX	8	11
Penta E	11	13
TH01	8	10
D12S391	20	21
D2S1338	17	21
FGA	22	22

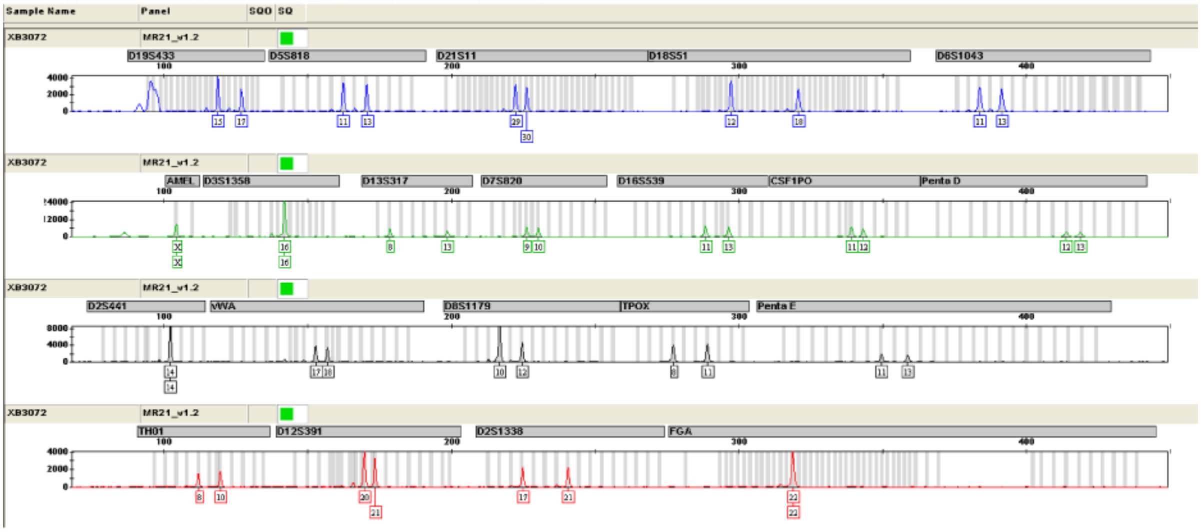

Supplementary Figure S16. The original blot images of Western blots were shown.

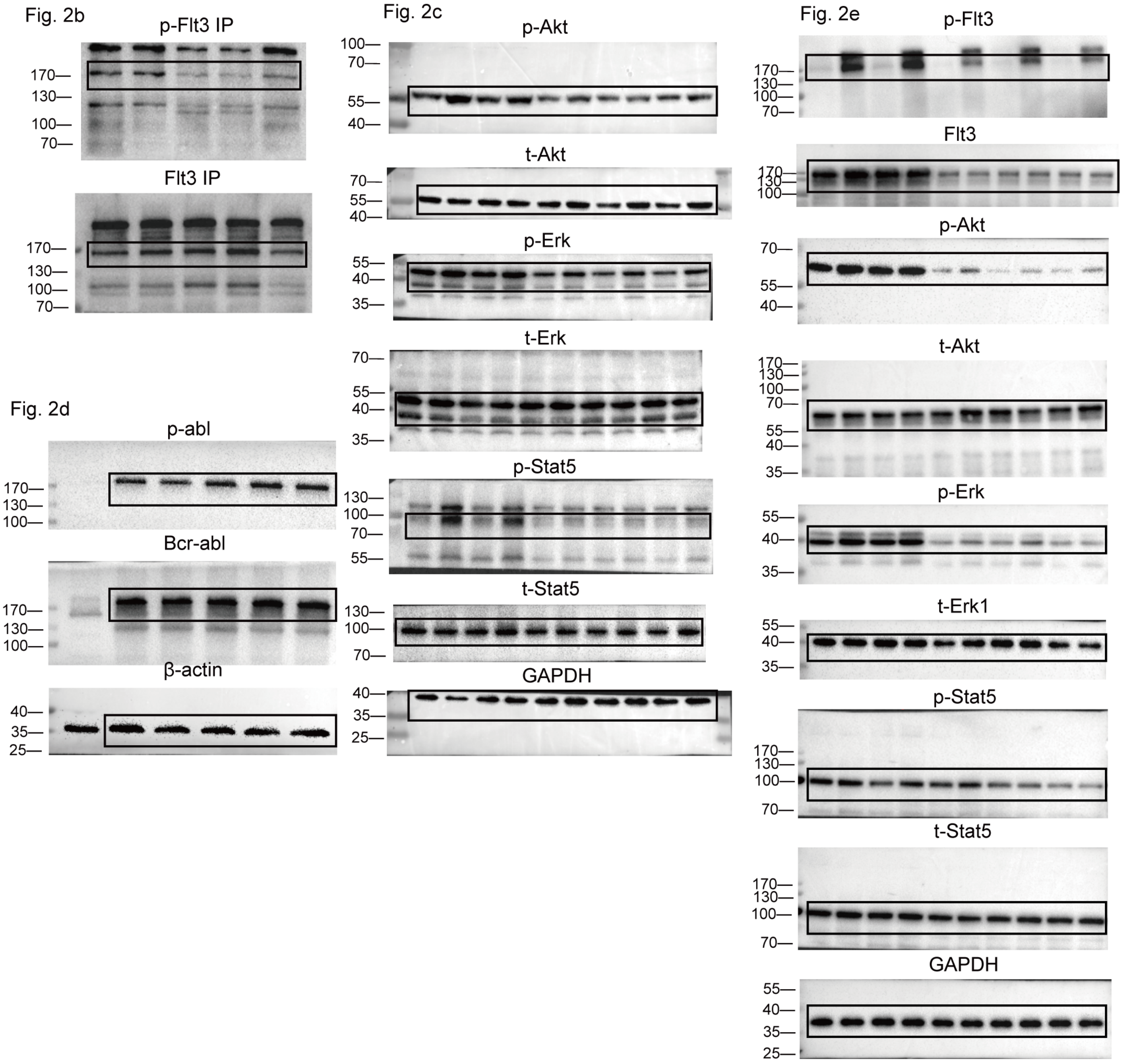

Supplementary Figure S17. The original blot images of Western blots were shown.

Supplementary Fig. S5

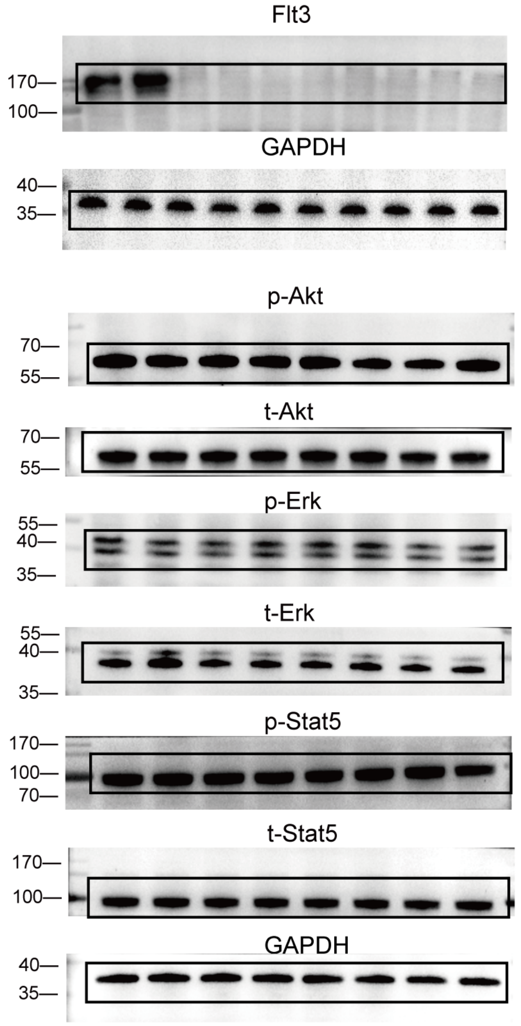

Supplementary Fig. S6

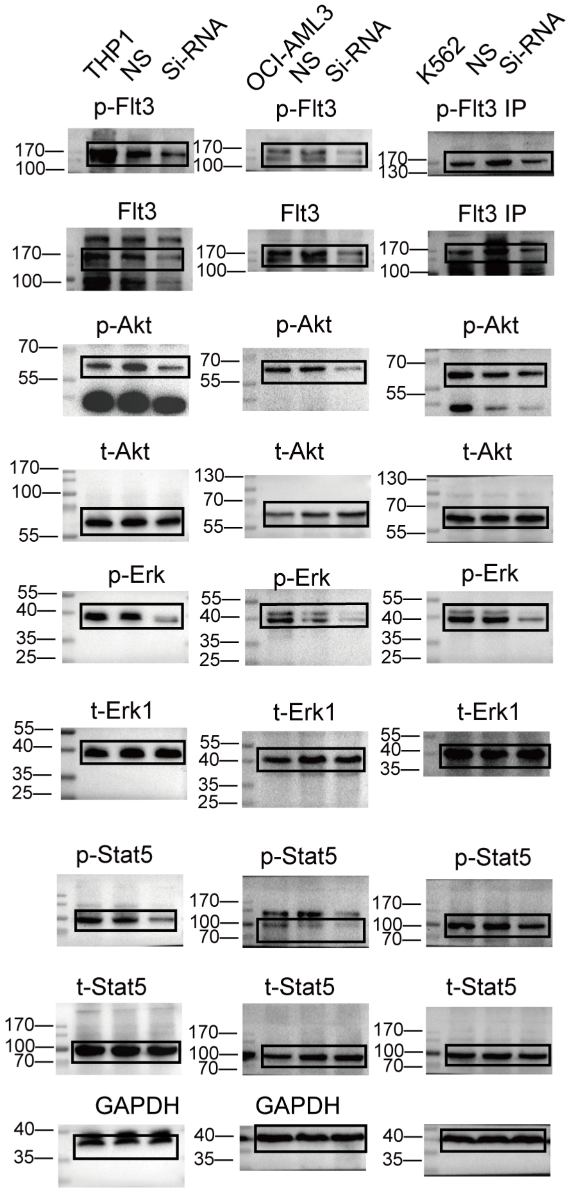

Supplementary Table S1. Summary of the sequencing results of mutant OCI-AML3 and Jurkat clones.

Sanger sequencing results of three candidate OCI-AML3 clones and two Jurkat clones carrying mutations within FLT3 exon 14 in single allele.

Clone	Mutation site	Genomic sequence change	Predicted translation change
Clone o35	Intron13/Exon14	73bp deletion	Disrupted intron/exon junction
Clone o166	Exon14	14bp deletion	Premature stop codon
Clone o253	Exon14	35bp deletion	Premature stop codon
Clone j105	Exon14	7bp deletion	Premature stop codon
Clone j212	Exon14	19bp deletion	Premature stop codon

Supplementary Table S2. Quality assessment of RNA-seq.

The RIN number was obtained using Agilent 2100. The high-quality reads were aligned to the human reference genome (NCBI Build 36.1) using SOAPaligner-v2.21 software (BGI). The matched reads were aligned to Human Refseq mRNA (NCBI). Abbreviations: RIN, RNA integrity number.

Sample	RIN	Total Reads	Total mapped reads (to reference genome)	Total mapped reads (to reference genes)
Clone kw1	9.9	11,253,376 (100.00%)	9,646,429 (85.72%)	8,359,576 (74.29%)
Clone kw2	9.9	12,212,959 (100.00%)	10,702,607 (87.63%)	9,339,282 (76.47%)

Clone kw3	10.0	11,943,694 (100.00%)	10,292,424 (86.17%)	8,939,708 (74.85%)
Clone k20	9.9	12,145,740 (100.00%)	10,958,316 (90.22%)	7,462,671 (61.44%)
Clone k114	9.6	11,733,634 (100.00%)	10,596,917 (90.31%)	7,099,063 (60.50%)
Clone k324	9.7	11,807,697 (100.00%)	10,663,747 (90.31%)	7,275,696 (61.62%)

Supplementary Table S3. The up-regulated genes related to the KEGG mRNA surveillance pathway and RNA degradation pathway in the FLT3 mono-allelic mutant group from RNA-seq analysis.

Abbreviations: RPKM, reads per kilobase of the target exon per million mapped reads; Mut, mutant group; Wt, wild-type group.

Symbol	RPKM						Fold change (Mut/Wt)	Probability
	Clone kw1	Clone kw2	Clone kw3	Clone k20	Clone k114	Clone k324		
UPF2	8.58	6.19	8.93	21.75	20.20	19.70	2.60	0.79
UPF3	9.62	8.47	8.36	16.44	17.41	17.25	2.92	0.82
SMG1	6.47	5.88	6.73	14.59	15.60	15.96	2.42	0.76
PPP2R5C	31.10	45.65	53.27	86.46	70.33	95.04	1.94	0.78
DIS3	4.89	4.09	4.94	12.85	12.94	10.87	2.62	0.76

EXOSC9	28.42	29.94	34.39	51.55	48.99	49.95	1.62	0.70
MPHOSPH6	12.59	23.81	26.88	35.47	46.74	41.01	1.95	0.76
CTNNAL1	11.29	11.66	16.04	23.84	26.26	27.12	1.98	0.75
SKIV2L2	22.71	19.56	25.64	44.91	48.53	47.02	2.07	0.78
TTC37	7.52	7.52	9.87	21.60	22.95	25.19	2.8	0.81
DMXL2	1.99	3.79	4.49	11.43	12.41	14.41	3.73	0.81
DHX29	10.63	10.83	13.20	23.15	25.86	24.59	2.12	0.77
DHX36	8.14	7.33	8.96	17.71	15.65	18.58	2.13	0.75
YTHDC2	4.86	4.28	4.50	9.45	10.75	10.58	2.26	0.71
CNOT6	8.73	7.43	7.98	16.21	18.07	15.68	2.07	0.73
MRPL42	14.88	13.65	17.02	30.69	30.15	31.56	2.03	0.76
LOC652276	6.15	6.44	6.34	17.33	12.89	12.30	2.25	0.74
RLIM	4.15	3.21	4.02	10.21	9.25	9.30	2.53	0.73
PABPC1L	9.83	8.48	11.30	18.69	15.85	27.02	2.08	0.75
PAN3	4.37	3.26	3.84	10.35	9.37	8.11	2.43	0.71

Supplementary Table S4. The RPKM values of the typical DEGs related to FLT3.

Abbreviations: RPKM, reads per kilobase of the target exon per million mapped reads; DEGs, differentially expressed genes.

Symbol	RPKM					
	Clone kw1	Clone kw2	Clone kw3	Clone k20	Clone k114	Clone k324

CCND3	234.76	378.87	350.58	194.10	123.55	238.34
BCL2L1	138.73	125.71	119.64	73.07	48.91	97.87
PIM2	168.41	182.37	173.22	94.95	57.28	83.39
XBP1	81.46	134.52	127.49	47.06	61.42	107.02
CLIC1	1411.37	1089.12	1041.25	800.37	571.13	761.28
TTK	11.35	9.71	12.47	32.68	35.12	22.02
MRPL12	140.18	167.59	166.33	87.72	110.73	89.53
ATAD2	12.95	12.04	14.62	29.56	25.86	34.91
MAPK6	14.10	14.81	16.88	31.09	29.51	34.50

Supplementary Table S5. Comparison between the authors' results and the Kim et al manuscript.

Gene symbol	Gene expression level with FLT3 haploinsufficiency	Gene expression level with FLT3 inhibition by TKIs
CCND3	Down	Down
XBP1	Down	Down
CLIC1	Down	Down
TTK	Up	Up
MRPL12	Down	Down
ATAD2	Up	Up
MAPK6	Up	Down

Supplementary Table S6. The primers used to amplify the possible off-target sites.

Abbreviations: Chr, chromosome; Pos, position.

Chr	Pos start	Pos end	Forward	Reverse
15	101493507	101493557	GGGCCACTCATTTG AATGTTGG	TTCAGAAATCCCACT GCTGG
1	49450182	49450237	AGCTCAGGGAAAGT TGCTACA	TCCCTCTTGCTGGAAA CTCC
22	23058918	23058972	TGAAATCTCAGCCT CCCAGG	ACACGTGTTCTCTTAC CATGCT
3	85511944	85512005	AGCCTAAGTCACCC ACAAATGT	CAAGGCCTATGAAAC GAGGGT
12	24408760	24408818	CCAAAACAGTACTT TGAGGGTGT	AAGCTGAGCTAAAAG GTGTCT
18	75410205	75410253	ACAGTGGTGTTATT TCTTCCTTAGA	TGGCCAAGACTCTTG AATCTGT
6	122489980	122490028	ACTCACAGCCTCTC TCAGACT	AAGGAAAAGGGCTGT GTTGT
12	63674904	63674964	AGTTTGTCCCAAAT ATTGCATGACA	TGAGTTTTTCCTAGGA GCCAGT
4	188406808	188406864	AACCAAAGGAGTGA CCCACC	CATTGCAATGGGCTA GGGGA
16	33974767	33974830	GGAGCACTTTGTGG	TGGCCTCAAAGCACT

			CCTAAG	CCTAAT
13	90025779	90025836	TCTTAGGGAATCTG AAACAGGGT	ACCTTACTTTAAGCTG CTTGTCAC
5	101244705	101244752	CCCACAGGCTTCAA ACCAAAG	GCTGCTTGTCACTAGG GGAG
11	21883628	21883685	GAGGGTGGTGAGGA TGAAGA	CTCCCTGAGCTCTGTA CAGC
13	38038152	38038209	TGGAATGCCCTTGA TGAATCCA	AGAGTGAATCTTAAT GTAAACTCTGGA
11	48806415	48806478	GAGATGGGGAGGTG TCAAGC	AGTGATGGCAATCCT AGCCA
5	19982489	19982534	TCACAGTAGGCCAC AAAGGTC	TGAGACATACAGTGA AAAAGCTGT
15	54723742	54723804	CCAAACAGATGGCA ACCTCC	CCTGCCTGGTGGAAG TGAAA
1	86008188	86008246	TGGGGGATCATCTC TGCCAA	TCTCTAGAAAGAATC TGTGCAGT
5	26983417	26983476	TGCTGTTGTGTCATG ATCTTTGA	AATCGCCCAGTGTTA GCCAA
21	20756108	20756157	AGCTAGGGAAAATG AAATGGGT	TGCAGTACATGGAGC AGCTG

Supplementary Table S7. The primers used for RT-PCR.

Gene	Forward	Reverse
FLT3	GTGACCGGCTCCTCAGATAA	TTGCGTTCATCACTTTTCCA
BCL2L1	GAGCTGGTGGTTGACTTTCTC	TCCATCTCCGATTCAGTCCCT
PIM2	TCCCGTGGAGTTGTCCATC	GGCACCAGAACCAAAATCAATG
CCND3	TACCCGCCATCCATGATCG	AGGCAGTCCACTTCAGTGC
PPP2R5C	GTAATAAAGCGGGCAGCAGG	CAAAGTCAAAGAGGACGCAACA
SMG1	TCCTTTCCTGAGAGCCCTAAAA	TGTTCTAGCAATGGACAACACG
UPF2	GGAGAAAACACCTAACATCACCA	CCTTGTCAGTGAAAATCCCAACT
UPF3	GCACAAAGATACCATGTGGATGA	GGTGCTGGACTGTCGTCAC
GAPDH	GAAACTGTGGCGTGATGGC	CACCACTGACACGTTGGCAG

Supplementary Methods

SiRNA knock-down assay

Non-specific control and FLT3 siRNA were purchased from Guangzhou RiboBio Co., Ltd. The sequences of FLT3 siRNA duplexes were 5′-GCA ACA GCU UAU GGA AUU A dTdT-3′ and 3’-dTdT CGU UGU CGA AUA CCU UAA U-5’. The target sequence was GCA ACA GCT TAT GGA ATT A. For siRNA knock-down assays, cells (1-2 × 106 cells per 100 μl) were transfected with 1 μm FLT3 siRNA or control siRNA by nucleofection applying programs T16 for K562, X01 for OCI-AML3, and V01 for THP-1, respectively. Following transfection, the cells were incubated for 48 hours and then were subjected to RT-PCR, immunoprecipitation and immunoblot assay.

Tyrosine kinase inhibition assay

Tyrosine kinase inhibitors, Sorafenib and Quizartinib, were purchased from Selleck and resolved in dimethyl sulfoxide (DMSO). Cells were incubated with Sorafenib or Quizartinib at various final concentrations for 72 hour before the CCK-8 viability assay. All experiments were performed in triplicate.

Mass spectrometry

Immunoprecepitates from wild-type K562 cells were resolved on 8% SDS-PAGE gels and then stained with Coomassie brilliant blue. Gels containing the possible targeted bands were manually cut and destained with 30% ACN/100 mM NH4HCO3. The gels were digested overnight in 12.5 ng/μl trypsin in 25 mM NH4HCO3. Then peptides were extracted three times with 60% ACN/0.1% TFA and dried completely by a vacuum centrifuge. Experiments were performed on a Q Exactive mass spectrometer that was coupled to Easy-nLC (Thermo Fisher Scientific). MS data was acquired using a data-dependent top10 method dynamically choosing the most abundant precursor ions from the survey scan (300–1800 m/z) for HCD fragmentation. MS/MS spectra were searched using MASCOT engine (Matrix Science, London, UK; version 2.2) against a non-redundant database UniProt (145758 sequences http://www.uniprot.org/).
